# Liquid Crystalline and Gel Properties of Luminescent Cyclometalated Palladium Complexes with Benzoylthiourea Ligands

**DOI:** 10.3390/gels9100777

**Published:** 2023-09-25

**Authors:** Theodora A. Ilincă, Monica Iliș, Marin Micutz, Viorel Cîrcu

**Affiliations:** 1 Department of Inorganic and Organic Chemistry, Biochemistry and Catalysis, University of Bucharest, 4-12 Regina Elisabeta Blvd., Sector 5, 030018 Bucharest, Romania; 2 Department of Analytical and Physical Chemistry, University of Bucharest, 4-12 Regina Elisabeta Blvd., Sector 5, 030018 Bucharest, Romania

**Keywords:** luminescent gels, palladium, liquid crystals, benzoylthiourea, lyotropic, metallogel, rheology

## Abstract

The design and development of new luminescent metallogels formed by cyclometalated palladium(II) complexes in protic solvents were investigated by a combination of differential scanning calorimetry (DSC), polarized optical microscopy (POM), and rheology. Cyclometalated palladium(II) complexes based on imine ligand and ancillary benzoylthiourea (BTU) ligand showed red emission in solid and gel states. The formation of a lyotropic liquid crystal phase was observed for the complex bearing shorter alkyl groups on the BTU ligand. This complex also behaved as a thermotropic liquid crystal that displays a monotropic smectic A phase (SmA). Dynamic rheology measurements (frequency sweep in the 5–90 °C range) of the 1-decanol solution of palladium(II) complexes highlighted their supramolecular self-association ability to generate 3D networks and form gels as a final result.

## 1. Introduction

The self-assembly of molecules can induce long-range order and liquid crystalline properties using either temperature (thermotropic liquid crystals) or solvents (lyotropic liquid crystals). When compounds show both behaviors, they are termed amphotropic [1]. In addition, molecules can display self-assembly in a specific solvent upon cooling of solutions to give stable gels in which the solvent molecules are entrapped in the supramolecular three-dimensional structures. The design and development of metallogels received increased attention in the past two decades due to the interesting optical, magnetic, and catalytic properties that could emerge [2]. Metallogels are a particular class of gels in which metal is coordinated to an organic ligand as part of the gel network [3]. Gels with metal nanoparticles adhered to its network are also known. To design supramolecular metallogels, two approaches could be used: (i) in situ metal–ligand coordination-driven formation of extended polymeric structure upon addition of metal ions, or (ii) design of discrete metal–ligand low molecular mass complexes as monomeric units for the immobilization of solvent molecules via multiple weak noncovalent interactions such as hydrogen bonding and π–π stacking exerted by ligands or various unconventional metal–metal interactions [4,5]. The aggregation of self-assembled palladium(II) complexes to produce stable metallogels has been recently reviewed [6]. A considerably lower number of metallogelators based on palladium(II) complexes were reported in literature despite their significant potential in catalysis or the development of multiresponsive metallogels. Cyclometalated palladium(II) complexes were found to be efficient low-molecular-mass metallogelators at low concentrations both for protic and aprotic solvents and for various ionic liquids [7]. On the other hand, palladium(II) complexes were extensively studied for their liquid crystalline properties (thermotropic and lyotropic), especially those based on cyclometalated structures, including calamitic, discotic, and polycatenar mesogens. Neutral organometallic mononuclear [8], dinuclear [9], and tetranuclear [10] palladium complexes, mostly having a cyclometalated imine ligand and an acetylacetonate-based co-ligand, have been shown to possess both thermotropic and lyotropic properties. In this work, we report the development of new palladium(II) complexes with benzoylthiourea ligands (BTU) that can combine the thermotropic and lyotropic behavior with the gelation capability in protic solvents to form stable luminescent gels. Moreover, these complexes show an interesting transition from lyotropic mesophase to a gel state, in addition to the conventional sol–gel transition at increasing temperatures. Generally, a useful physico-mechanical approach to studying materials, almost irrespective of their aggregation phase/state and composition, is rheology, which essentially deals with the flow and deformation phenomena of matter. Moreover, there is a relatively recent point of view according to which rheology is or must be about the characteristics of matter leading to a particular flow and deformation behavior [11]. Rheological studies performed on liquid crystals (LCs) date back more than five decades (see the very informative review of Porter and Johnson [12]). Fewer studies on low molecular weight LCs are published nowadays [13,14,15], while a significant amount of scientific research is conducted on polymer liquid crystals. Nevertheless, the development of rheological methods of investigation and new rheometers has made possible complex studies of the viscoelastic properties of any material at different frequencies (frequency sweep tests) and magnitudes of the applied external deformation/stress and under controllable temperature conditions. In this respect, the dynamic (oscillatory) rheology method allows the assessment of the viscoelastic behavior of a small sample of material based on the frequency and temperature dependencies of some quantities, such as viscoelastic moduli (storage modulus, loss modulus), loss tangent, or dynamic viscosity. In the present work, two newly synthesized compounds exhibiting liquid crystalline properties (**4-Pr** and **4-Bu**) were investigated in 1-decanol solution through dynamic rheology over a broad range of temperature (5–90 °C) and frequency (0.25–100.00 Hz), to study their ability of gel-formation upon both heating and cooling.

## 2. Results and Discussion

### 2.1. Synthesis of the Palladium(II) Complexes

**Scheme 1:**
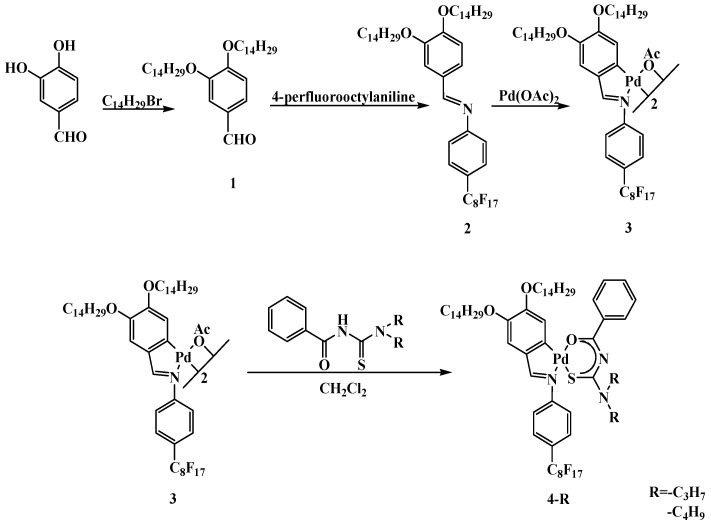
Synthesis of target palladium(II) complexes.

The synthesis pathway presented in Scheme 1 is based on the preparation of related palladium(II) complexes we reported earlier [16,17]. The reaction between 3,4-dihidroxybenzaldehide and bromotetradecane is the initial step in the synthesis, followed by the reaction with 4-perfluorooctylaniline to prepare the Schiff base **2**. This product was then employed in a cyclometalation reaction with palladium acetate to produce a high dinuclear palladium(II) complex **3** yield. In the final step, the dinuclear complex was employed to make the mononuclear palladium(II) complexes **4-R** by treating them with the appropriate benzoylthiourea derivatives.

### 2.2. NMR Spectroscopy

The ^1^H-NMR spectra are identical for the two **4-R** compounds, except for the decrease in integration of the protons of the alkoxy chains in the 1.76–1.38 ppm region. ^1^H-NMR spectroscopy showed the presence of the imine proton as a singlet at δ 8.17 ppm for both complexes. The signal around 9.00 ppm assigned to the NH group for uncoordinated BTU ligands was absent in the ^1^H-NMR spectra of the corresponding palladium(II) complexes because of ligand coordination in a deprotonated bidentate fashion similar to other cyclometalated palladium(II) complexes reported earlier. Even if the possibility of two different isomers exists in the solution, the ^1^H-NMR spectra of the mononuclear palladium(II) complexes display only one set of signals, implying that only one isomer is present in the solution. Another important feature of the ^1^H-NMR spectra of **4-R** is the magnetic nonequivalence of the protons of the alkyl groups from the N(Alk)_2_ moiety that give two sets of signals in the 3.90–0.88 ppm region due to restricted rotation along the C-N(Alk)_2_ bond. In the ^13^C-NMR spectra, the chemical shifts corresponding to the carbon from C=S were found at 172.87ppm and 172.91 ppm for **4-Pr** and **4-Bu**, respectively, while the values of the chemical shifts assigned to carbon atoms of the C=O groups were found at 170.16 and 170.12 ppm for **4-Pr** and **4-Bu**, respectively [18,19].

### 2.3. IR Spectroscopy

The precursors and the final compounds were analyzed through IR spectroscopy, and the bands and their assignments for these vibrational spectra are discussed further. While the ν(C=S) stretching vibration can be observed in the wide range at 720 cm^−1^ for the thiourea derivatives [20], the complexes show a lowering of the frequency by roughly 10 cm^−1^, from 720 cm^−1^ in the IR spectra of the uncoordinated thiourea ligands to 710 cm^−1^ in the IR spectra of complexes, indicating the coordination of the palladium ion through the S atom. The absence of the band at the range of 1610–1690 cm^−1^, corresponding to the typical range for ν(C=O) stretches in the IR spectra of complexes, is a consequence of the formation of the new products due to the coordination of the O atom to the palladium ion. The absence of the ν(NH) vibrations in the range 3100–3400 cm^−1^ in the IR spectra of the complexes also indicates the formation of these complexes by deprotonation of the NH group due to the coordination to the palladium center. The ν(CN) vibrations of the precursors are shifted in the IR spectra of complexes, meaning the absence of the hydrogen from the N atom from the BTU moiety. All this information suggests the absence of NH hydrogen between carbonyl and thiocarbonyl groups of the benzoyl thiourea moiety and the coordination in a bidentate fashion. In conclusion, the two mononuclear complexes have the metal center coordinated with the BTU ligands via O and S atoms and to the imine ligands via N and C atoms [21,22].

### 2.4. Thermal Stability of Palladium(II) Complexes

**Figure 1:**
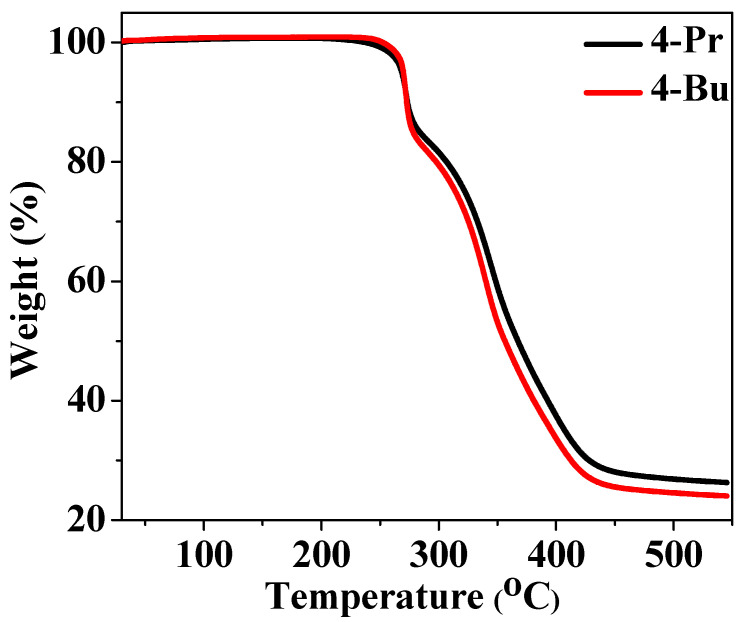
TG curves for palladium(II) complexes.

First, the thermal stability of the two mononuclear palladium(II) complexes was observed by thermogravimetric analysis (TG). The TG analysis showed that these materials are stable in the 25–250 °C interval, and their decomposition starts at temperatures higher than 250 °C. Figure 1 displays the TG curves for palladium(II) complexes. The TG curves do not show variations or mass loss in the 25–250 °C region, suggesting that the complexes do not contain small molecules (water or other solvents). The two palladium(II) complexes show decomposition in a single step up to 550 °C (26% residue for **4-Pr** and 24% for **4-Bu**, respectively).

### 2.5. Thermal Behavior of Palladium(II) Complexes

**Figure 2:**
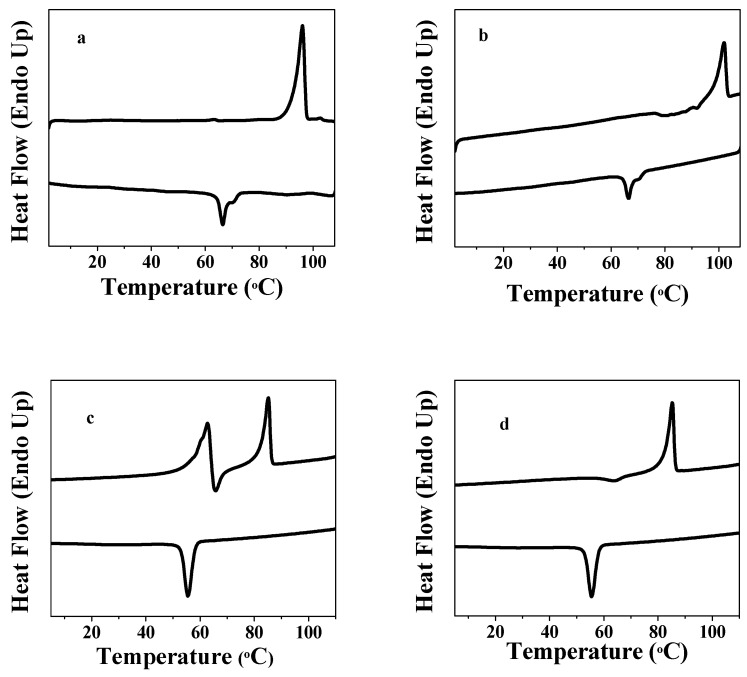
First and second DSC heating–cooling cycles for compound **4-Pr** (**a**,**b**) and **4-Bu** (**c**,**d**).

The thermal behavior of the palladium(II) compounds was investigated by a combination of DSC and POM methods to evaluate their stability and identify phase transitions. The transitions between a crystalline phase, a mesophase, and an isotropic phase are accompanied by a change in the enthalpy. The first heating run for both complexes exhibits a transition between two different crystalline phases followed by melting to isotropic phase at 101 °C for **4-Pr** and 82 °C for **4-Bu**. For **4-Pr**, POM observations indicated a mixture of liquid and crystals in the 92–101 °C temperature range, which changed completely to an isotropic phase above 101 °C. This latter transition corresponds to the small endothermic peak on the first DSC run (Figure 2a). On the subsequent DSC heating runs, the melting transition could be observed around 101 °C for **4-Pr** (Figure 2b). The melting temperatures are perfectly reproducible under heating–cooling cycle values reported in Table 1. The cooling runs show only a sharp peak assigned to the crystallization transition around 58 °C for **4-Bu** (Figure 2c). In contrast, **4-Pr** shows a broad asymmetric peak resulting from the overlapping of two different transitions, the isotropic state to a liquid crystal phase and crystallization (Figure 2a). The liquid crystalline phase was assigned based on its optical texture explored by POM.

**Figure 3:**
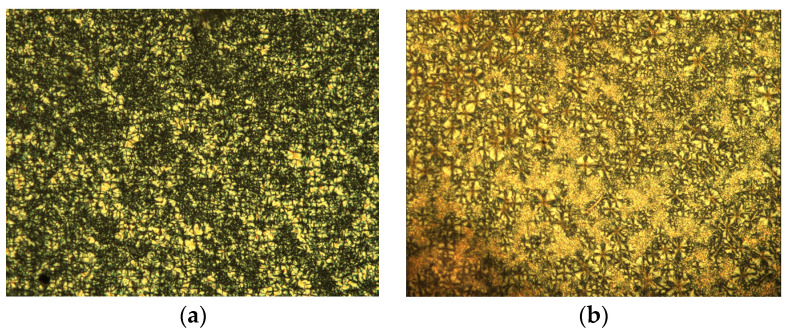
Pictures taken on cooling at the polarizing optical microscope show the optical textures of **4-Pr** at 69 °C (**a**) and 65 °C (**b**).

The characteristic optical texture observed by POM, when focal conic characteristics were observed in addition to several crystallization regions, was assigned to a monotropic SmA phase by analogy to the mesophases reported for related palladium(II) complexes (Figure 3). The increase in the number of carbon atoms from propyl to butyl in the BTU ligands leads to the destabilization of the SmA phase, indicating the delicate balance between the chain core and the microsegregation of the incompatible parts of mesogens (perfluoroalkyl and hydrocarbons groups).

This behavior is not entirely unexpected as the melting points and the transition temperature from isotropic to the monotropic SmA phase of the related palladium(II) complexes bearing N,N-dialkyl-N’-benzoylthiourea derivatives and Schiff base with dodecylalkoxy chains were strongly affected by the alkyl size. The related fluorine-free palladium(II) complexes show a significant decrease in the stability of the SmA phase with the increasing alkyl size of BTU ancillary ligand from propyl to butyl groups [23].

### 2.6. Spectroscopic Properties

**Figure 4:**
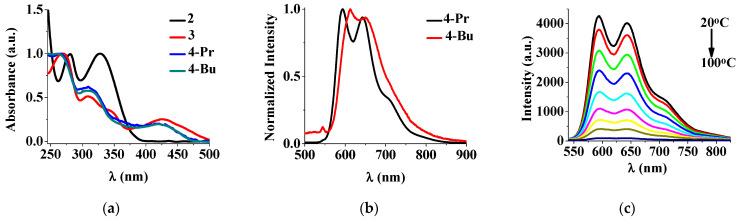
(**a**) The UV–VIS spectra of compounds **2–4** recorded in dichloromethane solution (conc. 10^−5^ M); (**b**) The normalized solid-state emission spectra of palladium(II) complexes (λ_exc_ = 365 nm); (**c**) Temperature-dependent emission spectra for **4-Pr** complex during heating from 20 °C to 100 °C.

The UV–Vis absorption spectrum of the free Schiff base ligand, together with the absorption and emission properties of palladium(II) complexes **4-R,** have been investigated, and the results are summarized in Table 2. Absorption and emission spectra of the fluorinated Schiff base and the respective cyclometalated palladium(II) complexes are shown in Figure 4.

**Figure 5:**
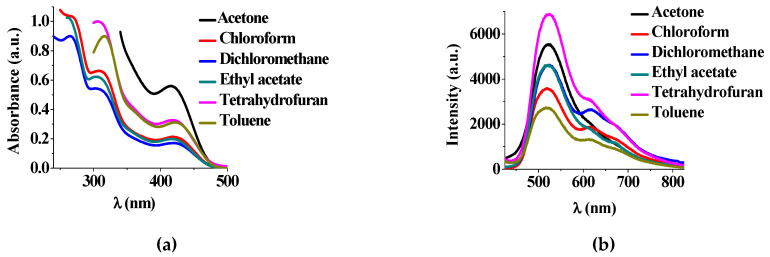
(**a**) The UV–VIS spectra of compound **4-Pr** recorded in different solvents; (**b**) The emission spectra of **4-Pr** in different solvents.

The UV–Vis absorption spectra (Figure 4a) in CH_2_Cl_2_ feature two intense absorption bands around 260 nm (ε > 28 × 10^3^/M^−1^ cm^−1^) and 310 nm (ε > 17 × 10^3^/M^−1^ cm^−1^) that are assigned to π–π* and n–π*—ligand-centered transition, based on similarities with the absorption spectra of the uncoordinated ligands, and a less intense absorption band at 420 nm (ε > 5.6 × 10^3^/M^−1^ cm^−1^) assigned to MLCT metal-to-ligand charge transfer (Table 2) [24]. The absorption spectra of **4-Pr** were recorded in different solvents to determine the effect of solvent polarity (Figure 5a). The lower-energy absorption band exhibits negative solvatochromic shifts (λ_max_ of the lowest energy absorption band is blue-shifted from 424 nm in toluene to 418 nm in ethyl acetate and 415 nm in acetone), showing this absorption as charge transfer in nature [25].

The solid-state emission of the two palladium(II) complexes (Figure 4b) shows two maxima around 590–610 and 640–650 nm, with a shoulder around 707 nm when the sample was irradiated at 365 nm. A slight change in the position of the maxima was observed on changing the carbon chain length (593 nm for **4-Pr** and 612 nm for **4-Bu**). The intensity of the yellow-orange emission varied amongst the two palladium complexes, which could be correlated with their lateral alkyl groups of the BTU ligands. A weaker intensity emission was observed for **4-Bu**, which could account for its higher structure flexibility due to the presence of longer butyl groups. When heating the sample to reach the liquid crystalline state, the emission intensity decreased rapidly (Figure 4b) because of both the influence of temperature and self-assembly in the liquid crystalline phase. The emission could be restored slowly, cooling the samples back to room temperature with a concomitant crystallization process.

The free Schiff base ligand **2** and complex **4-Bu** are nonemissive in dichloromethane solutions at room temperature. This phenomenon is common in palladium(II) complexes with weak-field ligands. The emissive excited states of most palladium(II) complexes can be easily quenched by the thermally accessible structurally distorted d-d excited state. Still, **4-Bu** shows moderate emission in solid state (Figure 4a). Consequently, the red-emission of **4-Bu** in the solid state could be ascribed to an aggregation-induced emission (AIE) phenomenon, also observed in palladium(II) complexes with thioamide-based pincer ligands [26,27]. In contrast, **4-Pr** showed intense emission in dichloromethane solution at room temperature with quantum yield Φ = 0.19% in non-degassed solvent estimated using [Ru(bpy)_3_]Cl_2_ complex as a standard. The emission spectrum of **4-Pr** in the solid state resembled that in the dichloromethane solution.

**Figure 6:**
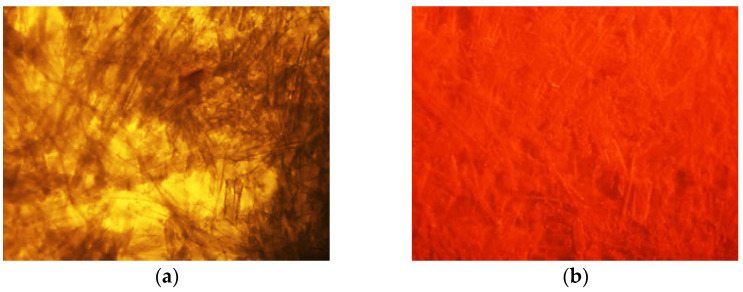
Pictures taken at the optical microscope showing compound **4-Pr** at room temperature (**a**) under normal light and (**b**) under UV light.

A blue shift of the emission maxima in the solution (520 nm in dichloromethane) was observed and compared with solid-state emission (593 nm). The emission properties of compound **4-Pr** were measured in various solvents to examine their effect on the emission spectra. As can be seen in Figure 5, the emission spectra maximin was around 520 nm with a shoulder of the same shape in all solvents. The solvent can influence the intensity and the position of the emission. When the polarity of the solvent decreases from acetone and ethyl acetate to toluene, the emission λ_max_ of **4-Pr** slightly red-shifts from 522 to 515 nm, similar to other cyclometalated palladium complexes [28]. In the absence of theoretical calculations and based on reports on luminescent palladium(II) complexes, the emission of **4-Pr** and **4-Bu** could be attributed to metal-perturbed ^3^π–π intraligand excited states [29]. The orange emission of the palladium complexes in the solid state was also confirmed by the picture taken with the optical microscope of the solid state in normal light and under UV light (Figure 6).

### 2.7. Gelation and Lyotropic Properties

**Figure 7:**
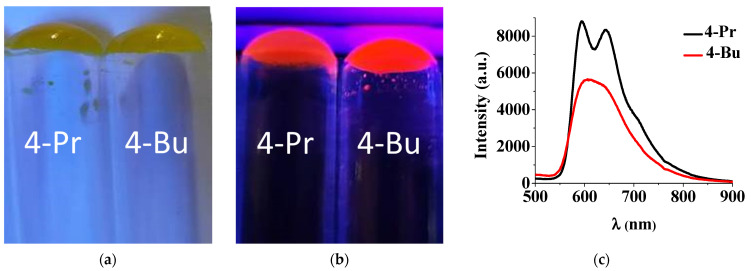
Photograph of gels formed by compounds **4-Pr** and **4-Bu** in DeOH in normal light (**a**) and by irradiation with UV light (λ_irr_ = 365 nm) (**b**). Emission spectra of palladium(II) complexes in gel state (**c**). (50 mg palladium complex in 1 mL DeOH).

The gel formation was observed during the crystallization procedure from the mixture of dichloromethane and ethanol for both complexes. Initially, the gelation ability of **4-Pr** was checked for seven different alcohols (methanol-MeOH, ethanol-EtOH, 1-propanol—n-PrOH, 2-propanol, i-PrOH, 1-butanol, BuOH, 1-octanol, OctOH and 1-decanol DeOH). The gelation test results are listed in Table 3. The compound was dissolved in the corresponding solvent by heating until the complete dissolution. The clear solution was then allowed to cool slowly to room temperature and form a gel state. The gelation properties were investigated according to the inversion of the test-tube method (Figure 7).

**Figure 8:**
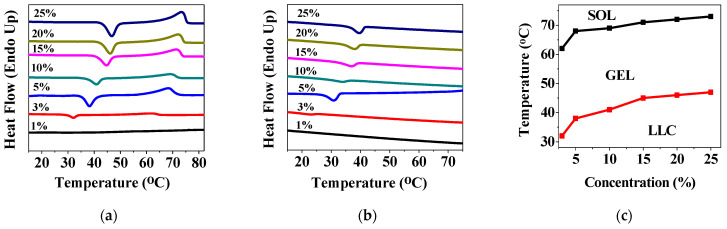
DSC trace of compound **4-Pr** at different concentrations during heating (**a**) and cooling (**b**). Phase diagram for **4-Pr** based on DSC data (**c**).

**Figure 9:**
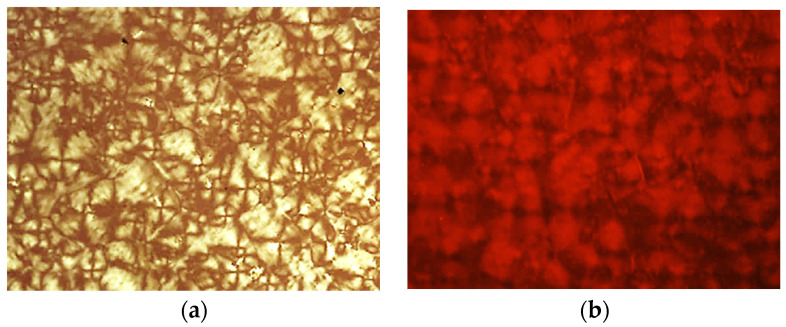
The lyotropic liquid crystal phase of compound **4-Pr** (5% gel) in normal polarized light (**a**) and by irradiation with UV light (**b**).

Further, we investigated the gel phase of **4-Pr** and **4-Bu** complexes 5% in 1-decanol via DSC. The sample was placed in an aluminum pan as a clear hot solution. The gelation process was observed to be thermoreversible for both complexes. Complex **4-Bu** shows only one transition during both heating and cooling runs assigned to sol–gel transition (Figure S11). In contrast, on cooling the solution of 5% **4-Pr** in 1-decanol, a clear transition at 33 °C was evidenced on the DSC trace (Figure 8b). On the basis of the optical texture seen at the polarizing microscope, this was assigned to the formation of a lyotropic mesophase (Figure 9). Because of their capacity to integrate a considerable amount of solvent, particularly nonpolar solvents, traditional thermotropic metallomesogens may also exhibit lyotropic features. Further, two different transitions were observed during heating: the first exothermic peak at 36 °C could be assigned to the transition from the lyotropic mesophase to the gel state, and the second endothermic peak to gel–sol transition (Figure 8a).

**Figure 10:**
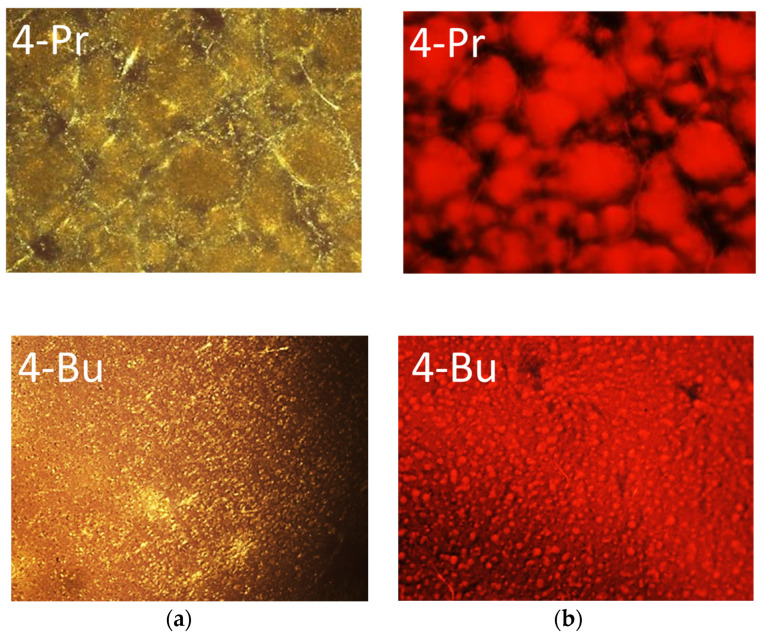
POM pictures showing the gel morphology for complexes **4-Pr** and **4-Bu** (**a**) and by irradiation with UV light, λ_irr_ = 365 nm (**b**). Images were taken during the gelation process at 25 °C for DeOH-based gels containing 50 mg/mL.

The transition that occurred around 36 °C was clearly observed by POM when well-defined crystalline structures were developed (Figure 10 and Figure S13). Several mixtures with various concentrations of **4-Pr**, ranging from 1% to 25% in 1-decanol, were investigated further by DSC. While at a concentration of 1%, the compound exists only in the sol state; with increasing concentration, both the gel and the lyotropic liquid crystal states were observed. The higher stability of the lyotropic phase was seen as the concentration of **4-Pr** increased from 3% to 25% (Figure 8c). The thermal parameters of these transitions were extracted from the DSC thermograms and are presented in Table 4.

DSC and POM experiments support the agglomeration of **4-Pr** and **4-Bu** after dissolution in alcohol. The X-ray structures of the related palladium(II) complexes reveal C-H^…^O (alkoxy chains) and C-H^…^π intermolecular interactions in their packing diagrams [21,23]. In addition, gelation of **4-Pr** and **4-Bu** is driven by agglomeration caused by noncovalent interactions, such as intermolecular C-F^…^H-C and hydrogen bonding, as well as van der Waals interactions. Furthermore, the presence of BTU auxiliary ligands could contribute to the observed gelation. The benzoylthiourea compounds are well-known to form inter- and intramolecular hydrogen bonding [30,31], and the gelation is also likely to be caused by alcohol solvent-mediated hydrogen-bonding interactions. Notably, the luminescence properties were preserved in the lyotropic phase and gel state, as presented in Figure 9, Figure 10 and Figures S12 and S13. No significant change in the position of emission maxima were found in the emission spectra of **4-Pr** and **4-Bu** in the gel state compared to the solid state (Figure 7c).

### 2.8. Assessment of Viscoelastic Behavior of 1-Decanol **4-Pr** and **4-Bu** Solutions

**Figure 11:**
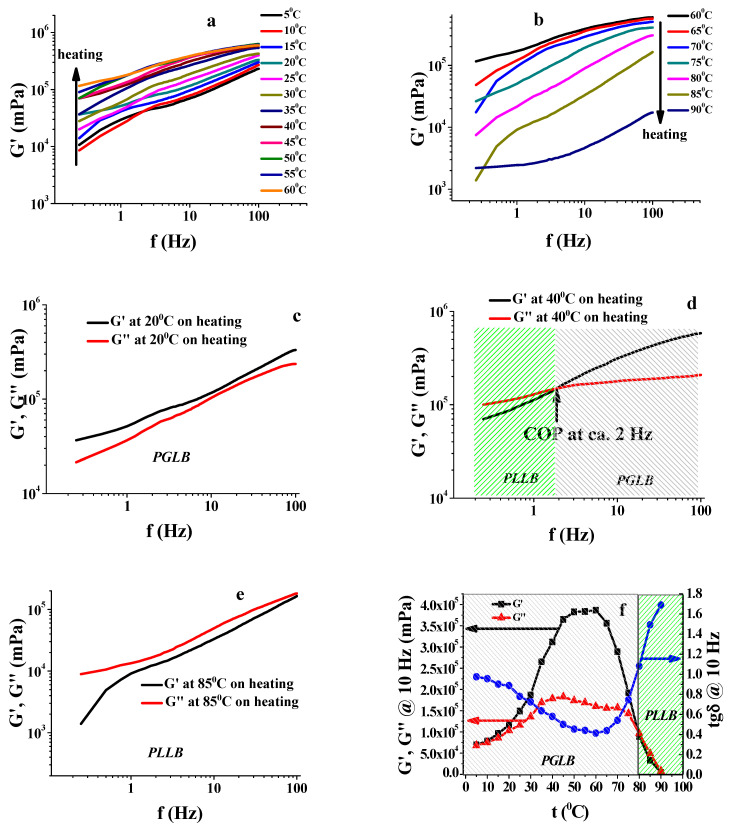
Rheograms of **4-Pr** in 1-decanol (50 mg/mL) taken during heating: storage modulus-frequency dependencies within 5–60 °C (**a**) and 60–90 °C (**b**); pairwise frequency dependencies of viscoelastic moduli revealing a PGLB behavior with no crossover point (**c**), both PGLB and PLLB behavior with a crossover point (**d**) and a PLLB behavior with no crossover point (**e**); viscoelastic moduli and loss tangent variation as a function of temperature considered at the same frequency of 10 Hz (**f**).

**Figure 12:**
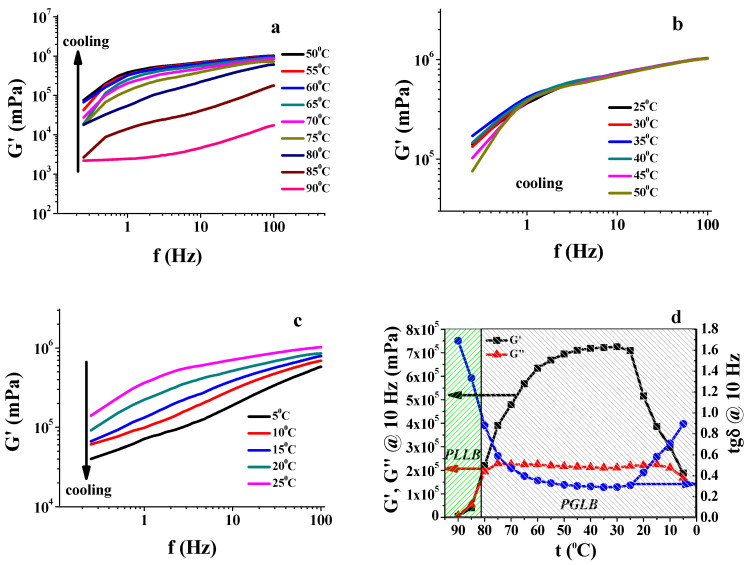
Rheograms of **4-Pr** in 1-decanol (50 mg/mL) recorded during cooling: storage modulus-frequency dependencies within 90–50 °C (**a**), 50–25 °C (**b**) and 25–5 °C (**c**); viscoelastic moduli and loss tangent variation on temperature considered at the same frequency of 10 Hz (**d**).

**Figure 13:**
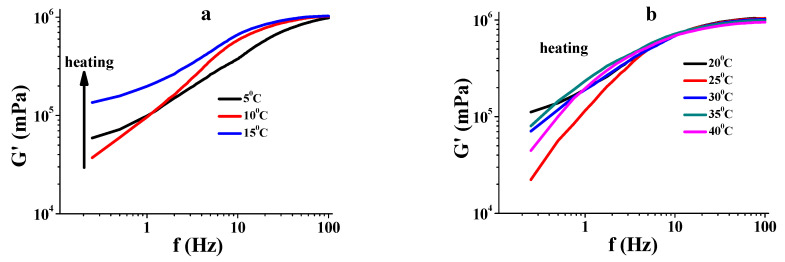

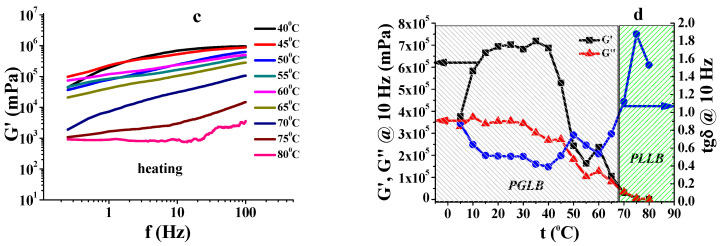
Rheograms of compound **4-Bu** dissolved in 1-decanol (50 mg/mL) acquired during heating from 5 to 80 °C: storage modulus-frequency dependencies within 5–15 °C (**a**), 20–40 °C (**b**) and 40–80 °C (**c**); viscoelastic moduli and loss tangent changes on temperature considered at the same frequency of 10 Hz (**d**).

**Figure 14:**
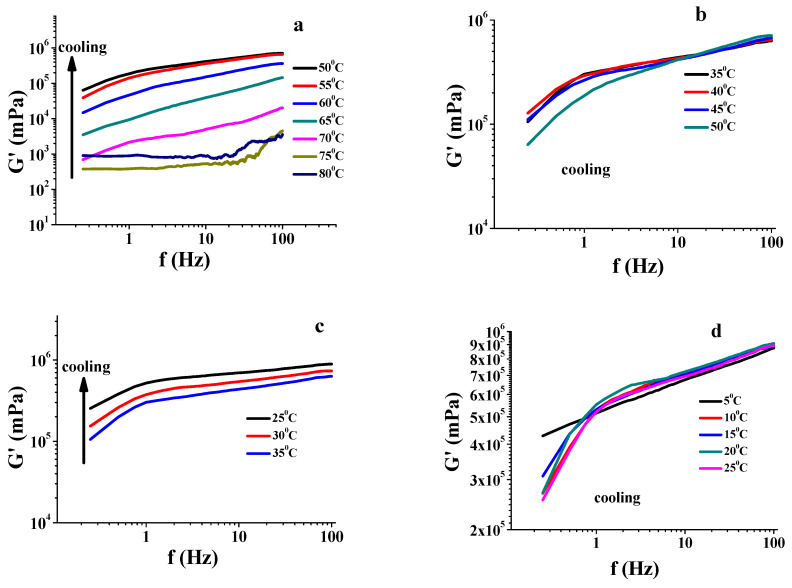

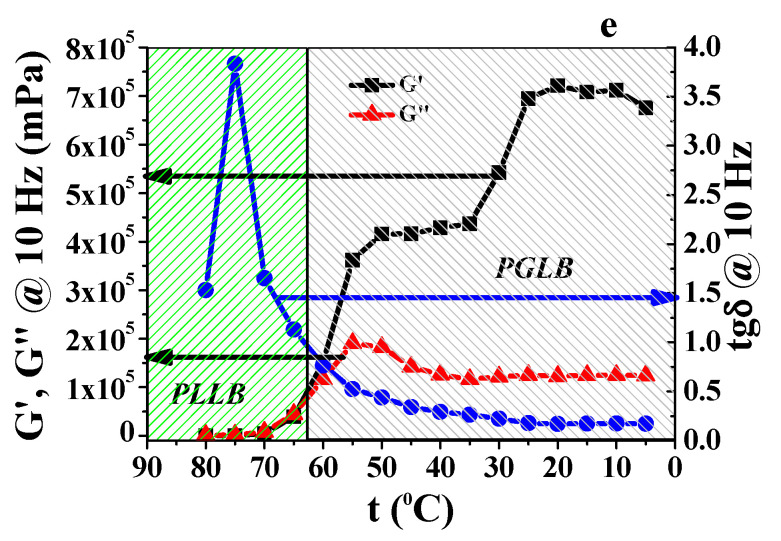
Rheograms of compound **4-Bu** dissolved in 1-decanol (50 mg/mL) recorded during cooling from 80 to 5 °C: storage modulus-frequency dependencies within 80–50 °C (**a**), 50–35 °C (**b**), 35–25 °C (**c**) and 25–5 °C (**d**); viscoelastic moduli and loss tangent changes on temperature considered at the same frequency of 10 Hz (**e**).

Prior to discussing the results of the viscoelastic behavior of the investigated systems, it is worth underlining that G′ measured for both solutions at all operational temperatures exhibited a continuous and specific increase in the frequency (f) of the applied deformation according to the general viscoelastic behavior postulated for the structured liquids where five relatively distinct regions can be delimited: viscous regime, transition to flow, rubber-like plateau, leathery state, and glassy state [14,32]. This particular tendency can be observed in the rheograms collected in Figure 11a,b, Figure 12a–c, Figure 13a–c and Figure 14a–d.

Because the squeezing deformation involved minimal vertically exerted oscillatory displacements (0.03–0.04 μm) compared with the sample thickness (300 μm), it is reasonable to suggest that the overall sample configuration/microstructure and its viscoelastic behavior are not affected by the deformation amplitude but only by the frequency of applying the external deformation and temperature. Such a general picture corresponds to a particular rheological testing regime called linear viscoelasticity. Mathematically speaking, this means that a stress τ_1_ induced by a strain ε_1_ may be linearly combined with a stress τ_2_ because of a strain ε_2_ into a final stress τ (as a sum of τ_1_ and τ_2_) generated by a total strain ε, as in Equation (1) following [11,33,34]:

(1) $$\tau =\tau (\varepsilon_{1}+\varepsilon_{2})=\tau (\varepsilon )=\tau_{1}+\tau_{2}$$

Alternatively, the strain varies linearly with stress. The experimental approach followed in this study (very small amplitudes of oscillatory shearing) is nondestructive for any material tested in this way [33,35].

On the other hand, the shape of *G”-f* dependences is strongly influenced by the specific system configuration/microstructure, which is determined by both solute–solvent interaction and solute–solute self-association at a certain temperature for a constant composition. Starting with *G′* (a measure of the amount of energy stored/recovered per deformation cycle and given by the ratio between the stress in phase with strain in an oscillatory deformation and the corresponding strain and *G”* (a measure of the amount of energy dissipated/lost per deformation cycle and given by the stress 90° out of phase with strain in an oscillatory deformation divided by the corresponding strain) and loss tangent (*tgδ = G”/G′,* where *δ* is phase angle) [33], two simple types of relationships can be inferred: (a) a material displaying a predominant storage behavior during deformation characterized by order of relationships *G′ > G″* and *tgδ* < 1, and (b) a material having a preponderant energy-dissipative behavior fairly characterized by *G′ < G″* and *tgδ* > 1. The first case involves a material that could possess predominantly gel-like behavior (PGLB), and the second with a material exhibiting largely liquid-like behavior (PLLB). For the limiting cases of pure rheological behavior, (a) *G′* has a positive and finite value while *G″* vanishes (*tgδ* becomes infinity) in a purely solid behavior (with storage component only) and, (b) *G″* has a positive and finite value while *G′* vanishes (*tgδ* becomes zero) in a purely liquid behavior (with loss component only). When *G′ = G″*, the two storage and loss components are balanced and describe the so-called gel point. This can frequently appear in the viscoelastic systems when the two rheograms *G′-f* and *G″-f* are crossed into a crossover point (COP) [36,37]. Regarding the COP, we considered its abscissa (expressed in Hz) in viscoelastic moduli-frequency plots.

The viscoelastic behavior of the compound **4-Pr** in 1-decanol solution is illustrated in detail in the rheograms in Figure 11 and Figure 12.

The sweep frequency tests during heating and cooling revealed two specific evolutions with temperature.

During heating from 5 °C to 90 °C, *G′-f* dependences continuously changed their places in the plots with temperature in a two-way manner:

- from 5 °C to 60 °C, these rheograms shifted to higher and higher values of *G′* almost over the entire frequency domain (Figure 11a);
- from 60 °C to 90 °C, the same type of dependences moved to lower and lower *G′* values considering the same frequency range (Figure 11b).

From a practical viewpoint, the picture just outlined mirrors a continuous and significant increase in the sample stiffness/rigidity (expressed by G′ values) as the temperature rose from 5°C to 60 °C followed by a substantial decrease in the sample stiffness when temperature elevated further to 90 °C. By taking into account *G″-f* rheograms (only selectively shown in Figure 1), their position with respect to that of *G′-f* traces displayed three distinct cases strongly influenced by the working temperature. In scenario (a), *G′-f* is located above *G″-f* (*G′ > G″*, *tgδ < 1*, PGLB) (Figure 11c), in (b) *G′-f* crosses *G″-f* (*COP* so that *G′ = G″* and *tgδ = 1*, with PGLB and PLLB on either side of COP) (Figure 11d), and in (c) *G′-f* is located below *G″-f* (*G′ < G″*, *tgδ > 1*, PLLB) (Figure 11e). At the same time, the value of G″ may express not only the ability of the system to flow (to dissipate energy during oscillatory deformation) but also the consistency of the sample via dynamic viscosity (*η_dyn_*) according to Equation (2) [31,32]:

(2) ηdyn=G″ω

where *ω* is the angular frequency (in rad/s) defined as *2∙π∙f*. Thus, since the most reliable experimentally obtained data (*G′* and *G″* values) in the range of 0.25–100 Hz are acquired for frequencies higher than 2–3 Hz, a very suggestive diagram containing the heating temperature evolution of the values of *G′*, *G″*, and *tgδ* at 10 Hz is illustrated in Figure 11f. The two rectangular regions clearly delineate the two viscoelastic characters—PGLB (left side) and PLLB (right side). Within the left-side area (5–80 °C), the gel-like behavior is enhanced in the temperature range of ca. 40–75°C (relied on the *G′* and *tgδ* values), reaching a plateau between about 45 °C and 60 °C. In fact, in this temperature region, the sample (decanol solution of **4-Pr**) took on stronger gel-like behavior, most likely due to a self-association of **4-Pr** in solution. Such a particular microstructure (between 40–75 °C) seems adequately supported by the DSC data on heating (Figure 8a), where the gel state of the same system was associated with the thermal domain of 36–62 °C. Moreover, as shown in Figure 11f, *G″* values follow more or less the same tendency as those of *G′*, which means that the consistency of the sample was substantially enhanced within the same relatively broad temperature range of 40–75 °C. These findings are noted in Table 1, where the values of dynamic viscosities at 10 Hz are inserted. The almost gel-like structure, however, completely disintegrated at the temperature above 80 °C when the liquid-like behavior became prevalent. More details regarding the COPs and the overall PGLB and PLLB of the decanol solution of **4-Pr** (together with the same characteristics for the compound **4-Bu** in the decanol solution) can be seen in Table S1.

During cooling from 90 to 5° C, *G′-f* dependences recorded for the 1-decanol solution of **4-Pr** also changed their places in the plots with temperature, but in a three-way manner (Figure 12a–c):

- from 90 °C to 50 °C, these rheograms shifted to higher and higher values of *G′* (at a decreasing rate) over the entire frequency domain (Figure 12a),
- from 50 °C to 25 °C, the same type of dependences kept almost indistinguishable/superimposed positions at the highest plateau *G′* values considering the same frequency range (Figure 12b) and, finally,
- from 25 °C to 5 °C, *G′-f* dependences displaced their positions to lower and lower *G′* values in the same frequency range (Figure 12c).

On the basis of the three peculiarities in the data shown in Table S1 and the temperature evolution of the G″ values taken at 10 Hz in Figure 12d, following the approach applied in heating, the compound **4-Pr** in decanol solution exhibited a similar effect of gel-formation when the system was gradually cooled down from 80 °C. Again, this transition from a solution with the **4-Pr** molecules randomly distributed into the solvent environment (90–80 °C) to an almost gel-like microstructure (PGLB) corresponds to favorable attractive interactions amongst solute molecules, with solute entities self-assembling into a large network encompassing the entire system volume. The strongest gel-like behavior was reached at 50–25 °C, below which the strength of the gel-like structure was progressively diminished (Figure 12d). This temperature domain of the strongest gel is in fairly good agreement with that detected through DSC during heating at 36–62 °C (Figure 8a). Interestingly, this temperature range of the gel state could not be seen on the DSC thermogram displayed during cooling when a sol state for the system progressively cooled to 33 °C (Figure 8b). The discrepancy could be explained by the temperature variation algorithm used in the experiments. Dynamic temperature variation in heating and cooling (at a constant rate) in the DSC trials and a steady-state manner of temperature change during the rheological investigation favored the formation of the gel state over the lyotropic mesophase. The viscoelastic behavior of the 1-decanol solution **4-Bu** resembles, to a certain extent, the behavior described for the similar solution of **4-Pr**.

The analysis of the **4-Pr** compound corroborated the experimental findings regarding the *G′-f* rheograms shown in Figure 13a–c with those collected in Table S1 (COPs, region of gel-like behavior, and dynamic viscosities taken at 10 Hz) and with those in Figure 13d (values of *G′*, *G″*, and *tgδ* at 10 Hz as a function of temperature). The viscoelasticity behavior of **4-Bu** during heating displayed the same ability of gel formation as a result of its supramolecular self-assembly in decanol solution in the temperature range of 5–65 °C, with the best-defined PGLB within 20–40 °C (where the values of loss tangent were the smallest). However, at temperatures beyond 65–70 °C, the gel-like behavior changed into an energy-dissipative behavior (PLLB), considering the values of *tgδ* (Figure 13d). Comparing the heating temperature range of the enhanced gel-like properties established by dynamic rheometry (20–40 °C) with the DSC data (15–40 °C for the gel-state existence) acquired for the same system (1-decanol solution of **4-Bu**) (Figure S11), the final results could be considered more than satisfactory, and the differences between them could be attributed to different methods of investigation, including the heating/cooling method adopted in DSC (at the rate of 10 °C/min) and the rheological measurements (temperature equilibrating time of 15 min at different programmed values).

A rheological study of the 1-decanol solution of **4-Bu** during cooling (from 80 °C to 5 °C) revealed a very interesting viscoelastic behavior. Again, taking into account the same rational route of analysis, the gel formation ability of this compound can be connected primarily to four different ways of shifting *G′-f* rheograms with temperature decrease, as illustrated in Figure 14a–d. By acquiring the values of *G′*, *G″*, and *tgδ* taken at 10 Hz, a useful picture of graphically plotted data shown in Figure 14e was obtained.

Thus, the gel-like state (60–5 °C, Figure 14e) was characterized by a stepwise increase in *G′* values taken at 10 Hz as temperature decreased and with two distinct plateaus. On the other hand, G*″* values at 10 Hz evolved differently with temperature decrease, reaching a maximum of around 55 °C. These two types of temperature dependence eventually led to a monotonically decreasing loss tangent as the temperature lowered. Even more interesting are the minimal values of loss tangent (below 0.2) compared with those found for the other analyzed gel-like configurations. Specifically, the gel-like structures appeared by cooling the 1-decanol solution of **4-Bu** and gained a stronger gel character (greater and greater ability of energy storage/recovery during oscillatory deformation exerted on the sample into the linear viscoelasticity domain). However, beyond this gel-enhancing effect, a careful inspection of Figure 14e provides valuable information: the gel state with the strongest gel character (highest G*′* values and smallest G*″* values) is compatible with the temperature decrease range of 25–5 °C, which is in an excellent agreement with the DSC data recorded for cooling with temperature of gel existence at below 26 °C (Table 4 and Figure S11). More details on the rheological investigation of **4-Pr** and **4-Bu** systems are collected in Table S1. A concluding remark concerning the viscoelastic behavior of the two 1-decanol solutions studied (heating/cooling) is that such data have to be cautiously analyzed when the working temperature is close to 5–10 °C because of the possible crystallization phenomena of 1-decanol (melting point at ca. 6 °C) coinciding with gel-formation/strengthening/weakening in both heating and cooling.

## 3. Conclusions

Cyclometalated palladium(II) compounds have excellent thermal stability (up to 250 °C), low melting points (101 °C for **4-Pr** and 82 °C for **4-Bu**), and are luminescent in solid form, while **4-Pr** is also luminescent in liquid crystalline state. Only **4-Pr** shows emission in dichloromethane solution with a quantum yield of 0.19% in a non-degassed solvent. As the polarity of the solvent increases, the absorption and emission bands of **4-Pr** change in a bathochromic manner. DSC and POM data demonstrate the existence of a short-range monotropic liquid-crystalline phase (between 68 and 70 °C) identified as SmA. Both complexes form luminescent stable gels in 1-decanol. In addition, a lyotropic phase was identified for **4-Pr**. By increasing the concentration of **4-Pr**, the stability of the lyotropic liquid crystal phase was observed to increase from 30 °C (3% **4-Pr** in 1-decanol) to 43 °C (25% **4-Pr** in 1-decanol). Dynamic rheology measurements (frequency sweep approach at different temperatures ranging from 5 to 90 °C) performed on the 1-decanol solution of compounds **4-Pr** and **4-Bu** underlined their ability of supramolecular self-association to generate 3D networks of solute in the tested solvent, with a final result in gel formation. The gel-like structures were developed on both heating and cooling. An apparently minor structural detail regarding the n-propyl or n-butyl moieties borne by the aminic nitrogen in **4-Pr** and **4-Bu**, respectively, led to quite different viscoelastic properties for the two compounds in their 1-decanol solution, including their gel-formation ability. Additionally, the DSC data confirmed the temperature domain for the presence of the gel-like state as detected rheologically. Because of its monotropic character and the working conditions, the lyotropic mesophase could not be clearly detected by rheology measurements.

## 4. Materials and Methods

### 4.1. Materials and Characterization Methods

All chemicals were used as they were provided. The ^1^H and ^13^C NMR spectra were obtained using a Bruker spectrometer (Bruker BioSpin NMR, Rheinstetten, Germany) set to 500 MHz and CDCl_3_ as the solvent. The ^1^H chemical shifts were calculated using the solvent peak location at 7.26 ppm. The sol–gel transition and the thermotropic behavior of the palladium complexes were observed by polarizing optical microscopy (POM) with a Nikon 50iPol microscope (Nikon Instruments, Melville, NY, USA), a Linkam THMS600 hot stage, and a TMS94 control processor (Linkam Scientific Instruments Ltd., Tadworth, UK). The samples were placed between two untreated glass slides. To study the lyotropic and gelation properties of palladium complexes, a drop of hot solution (80 °C) of corresponding palladium complexes in 1-decanol was placed on a glass plate, and a cover slide was placed on top. Then, the sample was observed during the cooling process to ambient temperature. Temperatures and enthalpies of transitions were measured using a Diamond DSC Perkin Elmer (Perkin Elmer, Boston, MA, USA). Following the encapsulation in aluminum pans, the compounds were examined at a 10 °C/min scanning rate. Each sample was subjected to three consecutive heating/cooling cycles. Emission spectra were acquired in solid and gel states using an OceanOptics QE65PRO spectrometer (Ocean Optics Inc., Orlando, FL, USA) linked to the microscope and a Nikon Intensilight excitation source. IR spectra were recorded on a Bruker Tensor V-37 spectrophotometer in KBr discs (Bruker Optics Inc., Billerica, MA, USA). A Jasco V-630 spectrophotometer (Jasco, Tokyo, Japan) was used to record the UV–Vis spectra in solution. The emission spectra in solution were measured in a 1 cm quartz cuvette placed in a holder connected to the OceanOptics QE65PRO detector via an optic fiber. The sample was irradiated by an LED light source (LLS-LED, OceanOptics, λ = 365 nm). Thermogravimetric analysis was performed using a TA Q50 TGA equipment or a Perkin Elmer STA 6000 instrument with alumina crucibles and nitrogen as a purging gas from room temperature up to 550 °C; the heating rate was 10 °C min^−1^. The gels formed by the palladium(II) complexes were identified by the inversion test. Dynamic rheology investigation (frequency sweep tests) was performed on solutions of **4-Pr** and **4-Bu** in 1-decanol (50 mg/mL) by employing a rheometer MFR 2100 (GBC, Dandenong, Victoria, Australia) equipped with a homemade jacket connected to a temperature controller (circulating water bath Lauda E100) in order to ensure a controllable temperature regime during the measurements. A volume of ca. 0.5 mL of specimen was placed on the bottom plate of the instrument, and then the assemblage of the parallel, circular upper (of 12.5 mm in radius) and bottom plates with the sample sandwiched between them was set to a constant operational gap of 300 μm. The random squeezing motion (pseudorandom noise shape) exerted vertically by the upper plate with an amplitude of 0.03–0.04 μm generates an associated force transmitted through the sample to the lower plate directly connected with a dedicated force sensor. A Fourier transform algorithm applied to the data (vertical displacement and force) continuously monitored during the measurements was used to obtain the values of both storage (G′) and loss modulus (G″) at 400 discrete frequencies simultaneously in the range of 0.25–100.00 Hz, with a step of 0.25 Hz. All the rheological measurements were performed in triplicate (with a relative standard deviation of max. 15%), with 30 scans for every single rheogram acquired and 15 min for the temperature equilibration at different programmed values.

### 4.2. Synthesis and Characterization of Palladium(II) Compounds

#### 4.2.1. Synthesis of Compound **1**

3,4-Di(tetradecyloxy)benzaldehyde was synthesized using a procedure described earlier in [38]. A mixture of 1-bromotetradecane (7.5 mL, 7 g, 25 mmol), 3,4-dihydroxybenzaldehyde (1 g, 7.2 mmol), and K_2_CO_3_ (7 g, 50 mmol) in 80 mL of MEK was heated under reflux for 72 h. After solvent removal, the obtained solid was purified by recrystallization from hot ethanol to yield 2.85 g (77%) of white crystalline product.

#### 4.2.2. Synthesis of Compound **2**

The imine ligand **2** was prepared by the condensation reaction between 4-(heptadecafluorooctyl)aniline (0.5 g, 1 mmol) and 3,4-ditetradecyloxybenzaldehyde (0.5 g, 1 mmol) in absolute ethanol. The mixture was heated under reflux for 2 h and then cooled to −25 °C to give the crude product yield 0.85 g (83%) of the white crystalline solid.

^1^H-NMR (500 MHz, CDCl_3_, 25 °C) δ (ppm) = 8.32 (s, 1H), 7.58 (m, 3H), 7.30 (m, 1H), 7.24 (s, 1H), 6.93 (d, J = 8.3 Hz, 1H), 4.08 (m, 4H), 1.90–1.82 (m, 4H), 1.49 (m, 4H), 1.42–1.21 (m, 41H), 0.88 (m, 6H).

^13^C-NMR (125MHz, CDCl_3_, 25 °C) δ (ppm) = 161.62, 155.79, 152.74, 149.52, 128.81, 127.89, 125.42, 124.75, 120.98, 112.28, 111.23, 69.19, 69.11, 31.94, 29.72, 29.68, 29.64, 29.63, 29.42, 29.41, 29.38, 29.20, 29.10, 26.04, 25.99, 22.70, 14.11.

IR (cm^−1^): 2918 (νCH_3_); 2874 (νCH_2_); 1578 (ν-C=N); 1516 (δNH); 1468 (νC-N); 1279 (νphenyl); 1239 (νphenyl); 1147 (νC-N); 1111 (νC-C, stretch (in-ring)); 1088 (νC-F); 1017 (νC-OCH_2_); 847 (δC-H); 722 (δC-C).

#### 4.2.3. Synthesis of Compound **3**

The imine ligand **2** (0.3 g, 0.29 mmol) was dissolved in dichloromethane (10 mL), and palladium acetate (0.065 g, 0.29 mmol) was added. The resulting mixture was stirred at room temperature for 24 h, then added ethanol and placed at −25 °C. The product was crystallized from a mixture of dichloromethane–ethanol to give the orange crystalline products, which were washed with cold ethanol and dried under vacuum. Yield was 0.330 g (96%) of orange crystalline product

IR (cm^−1^): 2919 (νCH_3_); 2850 (νCH_2_); 1587 (ν-C=N); 1530 (δNH); 1467 (νC-N); 1296 (νphenyl); 1274 (νphenyl); 1151 (νC-N); 1114 (νC-C, stretch (in-ring)); 1089 (νC-F); 941 (νC-OCH_2_); 848 (δC-H); 722 (δC-C).

#### 4.2.4. Synthesis of Compound **4-Pr**

The solid benzoylthiourea compound (9 mg, 0.035 mmol) and compound **3** (38 mg, 0.016 mmol) were dissolved in dichloromethane (10 mL). The resulting mixture was stirred at room temperature for 3 h. After solvent removal, the residue was purified on a silica gel chromatography column using eluent dichloromethane, followed by crystallization from dichloromethane/ethanol to yield a yellow solid of 23 mg 27%.

^1^H-NMR (500 MHz, CDCl_3_, 25 °C) δ (ppm) = 8.17 (s, 1H), 7.70 (d, J = 8.5 Hz, 2H), 7.67 (d, J = 7.6 Hz, 2H), 7.60 (d, J = 8.4 Hz, 2H), 7.36 (t, J = 7.3 Hz, 1H), 7.19 (t, J = 7.7 Hz, 2H), 7.05 (s, 1H), 7.03 (s, 1H), 4.16 (t, J = 6.7 Hz, 2H), 3.95 (t, J = 6.6 Hz, 2H), 3.90–3.84 (m, 2H), 3.81–3.75 (m, 2H), 1.98–1.90 (m, 2H), 1.90–1.83 (m, 2H), 1.79 (m, 2H), 1.72 (m, 2H), 1.52–1.20 (m, 44H), 1.04 (t, J = 7.4 Hz, 3H), 0.94 (t, J = 7.4 Hz, 3H), 0.88 (t, J = 6.4 Hz, 6H).

^13^C-NMR (125MHz, CDCl_3_, 25 °C) δ (ppm) =172.87, 170.16, 151.90, 151.67, 146.13, 138.38, 138.09, 130.99, 129.27, 127.72, 127.53, 123.79, 116.49, 115.82, 70.10, 68.66, 54.46, 53.42, 31.93, 30.92, 29.72, 29.67, 29.65, 29.45, 29.42, 29.38, 29.33, 29.09, 26.01, 25.99, 22.69, 21.29, 20.85, 14.11, 11.50, 11.47.

IR (cm^−1^): 2923 (νCH_3_); 2852 (νCH_2_); 1587 (ν-C=N); 1535 (ν_as_N-C-N); 1515 (δNH); 1468 (νC-N + νC-S); 1426 (νC=N + C=S); 1325 (ν_s_N-C-N + C-S); 1298 (νphenyl), 1268; 1227 (νN-CS); 1151 (νC-N); 1106 (νC-C, stretch (in-ring)); 1090 (νC-F); 1034 (νC-OCH_2_); 854 (δC-H); 710 (νPd-S); 657 (δC-C).

#### 4.2.5. Synthesis of Compound **4-Bu**

The solid benzoylthiourea compound (10 mg, 0.035 mmol) and compound **3** (38 mg, 0.016 mmol) were dissolved in dichloromethane (10 mL). The resulting mixture was stirred at room temperature for 3 h. The solvent was removed, and the residue was purified on a silica gel chromatography column using eluent dichloromethane, followed by crystallization from dichloromethane/ethanol to yield a yellow solid of 30 mg 66%.

^1^H-NMR (500 MHz, CDCl_3_, 25 °C) δ (ppm): 8.17 (s, 1H), 7.70 (d, J = 8.5 Hz, 2H), 7.68 (d, J = 7.7 Hz, 2H), 7.60 (d, J = 8.5 Hz, 2H), 7.36 (t, J = 7.3 Hz, 1H), 7.19 (d, J = 7.6 Hz, 2H), 7.05 (s, 1H), 7.02 (s, 1H), 4.13 (t, J = 6.7 Hz, 2H), 3.95 (t, J = 6.7 Hz, 2H), 3.91 (m, 2H), 3.82 (m, 2H), 1.92–1.83 (m, 4H), 1.79 (m, 2H), 1.68 (m, 2H), 1.52–1.22 (m, 48H), 1.05 (t, J = 7.4 Hz, 3H), 0.93 (t, J = 7.4 Hz, 3H), 0.88 (m, 6H).

^13^C-NMR (125MHz, CDCl_3_, 25 °C) δ (ppm): 172.91, 172.79, 172.58, 170.12, 151.95, 151.77, 151.31, 146.08, 138.36, 138.09, 130.98, 129.29, 127.73, 127.51, 127.08, 123.80, 116.38, 115.85, 70.12, 68.60, 52.39, 51.53, 31.93, 30.05, 29.72, 29.68, 29.65, 29.45, 29.43, 29.38, 29.34, 29.28, 29.14, 26.02, 25.99, 22.70, 20.37, 20.34, 14.12, 13.94, 13.81.

IR (cm^−1^): 2921 (νCH_3_); 2850 (νCH_2_); 1588 (ν-C=N); 1533 (ν_as_N-C-N); 1519 (δNH); 1467( νC-N + νC-S); 1422 (νC=N + C=S); 1327 (ν_s_N-C-N + C-S); 1297 (νphenyl), 1269; 1216 (νN-CS); 1144 (νC-N); 1110 (νC-C, stretch (in-ring)); 1088 (νC-F); 1025 (νC-OCH_2_); 851 (δC-H); 710 (νPd-S); 654 (δC-C).

### 4.3. Preparation of the Gels

For each sample, the appropriate amount of palladium(II) complexes **4-Pr** or **4-Bu** were dissolved in 0.1 mL of the corresponding alcohol. The compound **4-Pr** was also studied in different concentration ranges from 1% to 25% in 1-decanol. The inversion tube test was used to confirm the stability of the gels. The gels were investigated for their sol–gel transition temperatures by DSC, POM, and rheology.

## Figures and Tables

**Table 1:** Thermal parameters for the new compounds.

Compound	Transition, T/°C (ΔH/kJ mol^−1^)
**2**	Cr 68 (62.9) Iso 58 (−53.85) Cr
**4-Pr**	Cr_1_ 92 (63.7) Cr_2_ 101 (0.4) Iso 70 * SmA 68 (−19.6) Cr
**4-Bu**	Cr_1_ 57 (57.9) Cr_2_ 82 (31.3) Iso 58 (−25.8) Cr

Cr_,_ Cr_1,_ Cr_2_—crystalline phases; SmA—smectic A phases; Iso—isotropic phase. * transition detected by POM.

**Table 2:** Absorption and emission data for the Pd(II) complex **4-Pr** in dichloromethane solution (concentration 10^−5^ M) and solid state.

Compound	Absorption, λ_max_/nm (ε × 10^−3^/M^−1^ cm^−1^)	Emission, λ_em_/nm		
		Solution (λ_exc_/nm; Φ%)	Solid State	Gel
**2**	280 (33.1), 328 (33.3)	-	-	-
**3**	271 (30.8), 310 (15.7), 345 (10.6), 427 (7.7)	-	-	-
**4-Pr**	266 (28.0), 310 (17.1), 424 (5.6)	520 (365/0.19)	593, 642, 707 (sh)	595, 643
**4-Bu**	260 (36.6), 309 (22.8), 420 (7.7)	Not detected	612, 651	606

**Table 3:** Gelation properties of compound **4-Pr** in various alcohol solvents at 25 °C.

Solvent	Gelation Properties
MeOH	I
EtOH	PG
n-PrOH	G
i-PrOH	G
BuOH	G
OctOH	G
DeOH	G

I—insoluble, PG—partial-gel, G—gel.

**Table 4:** Thermal parameters for mixtures of **4-Pr** and **4-Bu** in 1-decanol.

Compound	Concentration (%)	Transition, T/°C (ΔH/Jg^−1^)
**4-Pr**	1	-
**4-Pr**	3	LLC 30 (−0.83) gel 58 (0.45) sol 25 (−0.36) LLC
**4-Pr**	5	LLC 36 (−1.65) gel 62 (1.37) sol 33 (−1.11) LLC
**4-Pr**	10	LLC 37 (−2.72) gel 60 (2.99) sol 36 (−1.02) LLC
**4-Pr**	15	LLC 41 (−3.98) gel 63 (5.08) sol 39 (−1.69) LLC
**4-Pr**	20	LLC 43 (−4.72) gel 65 (4.57) sol 40 (−1.90) LLC
**4-Pr**	25	LLC 43 (−5.38) gel 67 (6.30) sol 41 (−2.49) LLC
**4-Bu**	5	gel 40 (2.47) sol 26 (−1.86) gel

LLC—lyotropic liquid crystal.

## Data Availability

Not applicable.

## Supplementary Materials

The following supporting information can be downloaded at: https://www.mdpi.com/article/10.3390/gels9100777/s1, Figure S1: ^1^H-NMR spectrum for compound **2**, Figure S2: ^13^C-NMR spectrum for compound **2**, Figure S3: IR spectrum for compound **2**, Figure S4: IR spectrum for compound **3**, Figure S5: ^1^H-NMR spectrum for compound **4-Pr**, Figure S6: ^13^C-NMR spectrum for compound **4-Pr**, Figure S7: IR spectrum for compound **4-Pr**, Figure S8: ^1^H-NMR spectrum for compound **4-Bu**, Figure S9: ^13^C-NMR spectrum for compound **4-Bu**, Figure S10: IR spectrum for compound **4-Bu**, Figure S11: DSC trace for **4-Bu** 5% in 1-decanol, Figure S12: Lyotropic liquid crystal phase of compound **4-Pr** (15% gel) (a) under normal light and (b) under UV light at different temperatures, Table S1: Crossover points, relationships between the viscoelastic moduli and dynamic viscosities (at 10 Hz) for the compounds **4-Pr** and **4-Bu** (dissolved in 1-decanol) rheologically studied, Figure S13: Gel morphology revealed by POM for compound **4-Pr** in (a) natural light and (b) under UV light (images taken during the gelation process at 25 °C for DeOH-based gels containing 50 mg/mL).

## References

[1] Tschierske C. (2002). Amphotropic liquid crystals. Curr. Opin. Colloid Interface Sci..

[2] Wu H., Zheng J., Kjøniksen A.-L., Wang W., Zhang Y., Ma J. (2019). Metallogels: Availability, Applicability, and Advanceability. Adv. Mater..

[3] Dastidar P., Ganguly S., Sarkar K. (2016). Metallogels from Coordination Complexes, Organometallic, and Coordination Polymers. Chem. Asian J..

[4] Tam A.Y.-Y., Yam V.W.-W. (2013). Recent advances in metallogels. Chem. Soc. Rev..

[5] Liu Z., Zhao X., Chu Q., Feng Y. (2023). Recent Advances in Stimuli-Responsive Metallogels. Molecules.

[6] Ganta S., Chand D.K. (2018). Discrete and Polymeric Self-assembled Palladium(II) Complexes as Supramolecular Gelators. Chem. Asian J..

[7] Tu T., Bao X., Assenmacher W., Peterlik H., Daniels J., Dötz K.H. (2009). Efficient Air-Stable Organometallic Low-Molecular-Mass Gelators for Ionic Liquids: Synthesis, Aggregation and Application of Pyridine-Bridged Bis(benzimidazolylidene)–Palladium Complexes. Chem. Eur. J..

[8] Usol’tseva N., Espinet P., Buey J., Serrano J.L. (1997). Liquid-crystalline behaviour of di- and mono-palladium organyls: Two ways of lyomesophase formation. J. Mater. Chem..

[9] Usol’tseva N., Espinet P., Buey J., Praefcke K., Blunk K. (1997). Lyomesomorphic behaviour of selected di-palladium organyls in hydrocarbons. Mol. Cryst. Liq. Cryst..

[10] Usolt’seva N., Praefcke K., Singer D., Gündogan B. (1994). Lyotropic phase behaviour of disc-shaped tetra-palladium organyls in apolar solvents. Liq. Cryst..

[11] Malkin A.Y., Isayev A. (2017). Introduction. Rheology: Subject and goals. Rheology: Concept, Methods, and Applications.

[12] Porter R.S., Johnson J.F., Eirich F.R. (1967). The rheology of liquid crystals. Rheology. Theory and Applications.

[13] Ramos C.L., Zapotocky M., Lubensky T.C., Weitz D.A. (2002). Rheology of defect networks in cholesteric liquid crystals. Phys. Rev. E.

[14] Mezzenga D.R., Meyer C., Servais C., Romoscanu A.I., Sagalowicz L., Hayward R.C. (2005). Shear rheology of lyotropic liquid crystals: A case study. Langmuir.

[15] Marenduzzo E.D., Orlandini E., Yeomans J.M. (2007). Hydrodynamics and rheology of active liquid crystals: A numerical investigation. Phys. Rev. Lett..

[16] Ilis M., Batalu D., Pasuk I., Cîrcu V. (2017). Cyclometalated palladium(II) metallomesogens with Schiff bases and N -benzoyl thiourea derivatives as co-ligands. J. Mol. Liq..

[17] Iliş M., Micutz M., Cîrcu V. (2017). Luminescent palladium(II) metallomesogens based on cyclometalated Schiff bases and N-benzoyl thiourea derivatives as co-ligands. J. Organomet. Chem..

[18] Iliş M., Micutz M., Pasuk I., Staicu T., Cîrcu V. (2017). Synthesis and liquid crystalline properties of novel fluorinated N-benzoyl thiourea compounds. Effect of perfluoroalkyl chains on the thermal behavior and smectic phases stability. J. Fluor. Chem..

[19] Imrich J., Bušová T., Kristián P., Džara J. (1994). Synthesis and the ^13^C NMR spectra of N,N−disubstituted benzoylthioureas and their seleno and oxo analogues. Chem. Pap..

[20] Saeed A., Khurshid A., Bolte M., Fantoni A.C., Erben M.F. (2015). Intra- and intermolecular hydrogen bonding and conformation in 1-acyl thioureas: An experimental and theoretical approach on 1-(2-chlorobenzoyl)thiourea. Spectrochim. Acta A Mol. Biomol. Spectrosc..

[21] Tenchiu A.C., Iliş M., Dumitraşcu F., Whitwood A.C., Cîrcu V. (2008). Synthesis, characterization and thermal behaviour of ortho-metallated Pd(II) complexes containing N-benzoylthiourea derivatives. Polyhedron.

[22] Iliş M., Micutz M., Dumitraşcu F., Pasuk I., Molard Y., Roisnel T., Cîrcu V. (2014). Enhancement of smectic C mesophase stability by using branched alkyl chains in the auxiliary ligands of luminescent Pt(II) and Pd(II) complexes. Polyhedron.

[23] Cîrcu V., Mănăilă-Maximean D., Roşu C., Iliş M., Molard Y., Dumitraşcu F. (2009). Mesomorphic behaviour and TSDC measurements of ortho-metallated palladium(II) and platinum(II) complexes with S,O-donor co-ligands. Liq. Cryst..

[24] Lai S.-W., Cheung T.-C., Chan M.C.W., Cheung K.-K., Peng S.-M., Che C.-M. (2000). Luminescent Mononuclear and Binuclear Cyclometalated Palladium(II) Complexes of 6-Phenyl-2,2′-bipyridines: Spectroscopic and Structural Comparisons with Platinum(II) Analogues. Inorg. Chem..

[25] Chow P.-K., Cheng G., Tong G.S.M., Ma C., Kwok W.-M., Ang W.-H., Chung C.Y.-S., Yang C., Wanga F., Che C.-M. (2016). Highly luminescent palladium(II) complexes with sub-millisecond blue to green phosphorescent excited states. Photocatalysis and highly efficient PSF-OLEDs. Chem. Sci..

[26] Kuwabara J., Ogawa Y., Taketoshi A., Kanbara T. (2011). Enhancement of the photoluminescence of a thioamide-based pincer palladium complex in the crystalline state. J. Organomet. Chem..

[27] Das D., Kannan S., Kumar M., Sadhu B., Kumbhare L.B. (2022). Synthesis, photophysical properties and catalytic activity of Ƙ3-SCS pincer palladium (II) complex of N,N′-di-tert-butylbenzene-1,3-dicarbothioamide supported by DFT analysis. Inorg. Chim. Acta.

[28] Dalmau D., Urriolabeitia E.P. (2023). Luminescence and Palladium: The Odd Couple. Molecules.

[29] Chow P.-K., To W.-P., Low K.-H., Che C.-M. (2014). Luminescent Palladium(II) Complexes with π-Extended Cyclometalated [R-C^N^N-R′] and Pentafluorophenylacetylide Ligands: Spectroscopic, Photophysical, and Photochemical Properties. Chem. Asian J..

[30] Yang W., Zhu W., Zhou W., Liu H., Fan J. (2012). Hydrogen bonding interactions in two isomers of fluorobenzoylthioureas and their absorption spectra. J. Fluor. Chem..

[31] Staicu T., Ilis M., Circu V., Micutz M. (2018). Influence of hydrocarbon moieties of partially fluorinated N-benzoyl thiourea compounds on their gelation properties. A detailed rheological study of complex viscoelastic behavior of decanol/N-benzoyl thiourea mixtures. J. Mol. Liq..

[32] Barnes H.A., Barnes H.A. (2000). Linear viscoelasticity and time effects. A Handbook of Elementary Rheology.

[33] Song K.-W., Kuk H.-Y., Chang G.-S. (2006). Rheology of concentrated xanthan gum solutions: Oscillatory shear flow behavior. Korea Aust. Rheol. J..

[34] Macosko C.W., Macosko C.W. (1994). Linear Viscoelasticity. Rheology: Principles, Measurements and Applications.

[35] Sadeghi-Mehr A., Raudsepp P., Brüggemann D.A., Lautenschlaeger R., Drusch S. (2018). Dynamic rheology, microstructure and texture properties of model porcine meat batter as affected by different cold-set binding systems. Food Hydrocoll..

[36] Ferry J.D., Ferry J.D. (1980). Illustrations of viscoelastic behavior of polymeric systems. Viscoelastic Properties of Polymers.

[37] Bernreitner K., Neiβl W., Gahleitner M. (1992). Correlation between molecular structure and rheological behaviour of polypropylene. Polym. Test..

[38] Yoo Y., Im J., Han B., Lee M., Choi M. (2001). Synthesis, Molecular Structure and Mesomorphic Phase Behavior of η^1^-Benzylideneaniline Palladium(II) Complexes. Bull. Korean Chem. Soc..

