# Supplementary material for: Liquid Crystalline and Gel Properties of Luminescent Cyclometalated Palladium Complexes with Benzoylthiourea Ligands
Source: Gels. 2023 Sep 25;9(10):777. doi: 10.3390/gels9100777 (PMC10606689; doi:10.3390/gels9100777)
Supplement: Supplementary file 1 [file gels-09-00777-s001.zip › gels-2616744-supplementary.pdf]

Supplementary Material

Liquid crystalline and gel properties of luminescent cyclometalated palladium complexes with benzoylthiourea ligands

Theodora A. Ilincă [1], Monica Iliș [1], Marin Micutz [2] and Viorel Cîrcu [1,*]

[1] Department of Inorganic and Organic Chemistry, Biochemistry and Catalysis, University of Bucharest, 4-12 Regina Elisabeta Blvd., Sector 5, 030018 Bucharest, Romania;
[2] Department of Analytical and Physical Chemistry, University of Bucharest, 4-12 Regina Elisabeta Blvd., Sector 5, 030018 Bucharest, Romania; micutz@gw-chimie.math.unibuc.ro
* Correspondence: viorel.circu@chimie.unibuc.ro

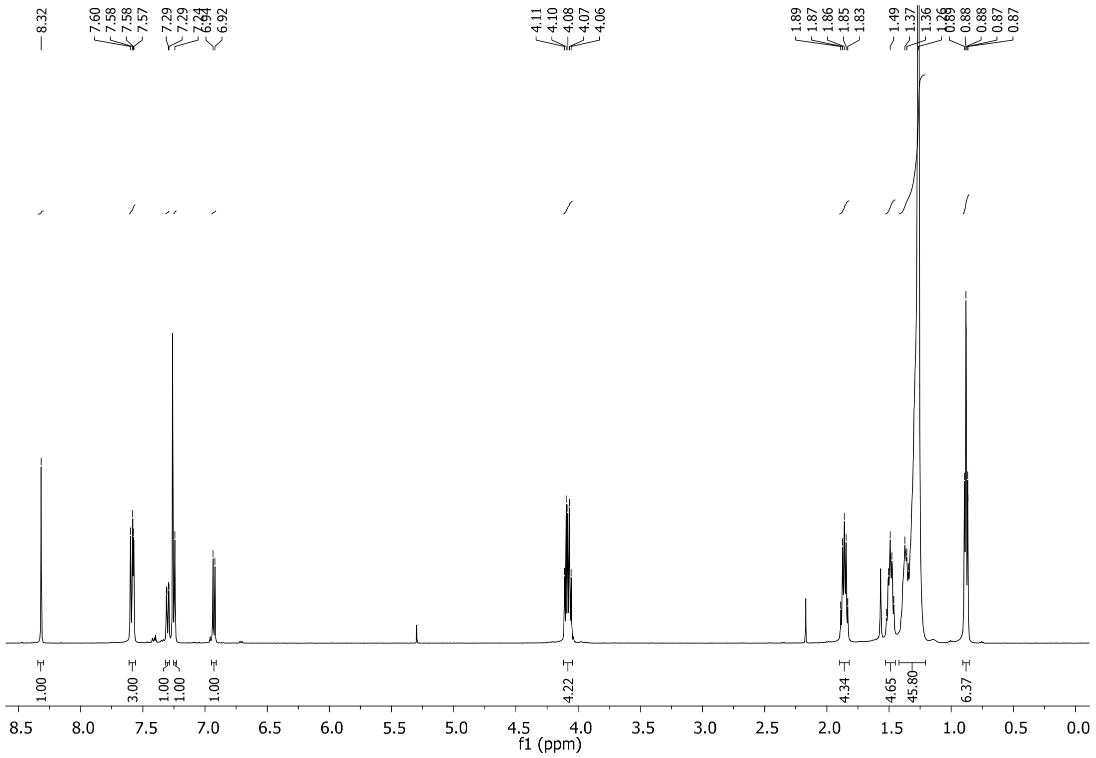

Figure S1. ^{1}H-NMR spectrum for compound **2**.

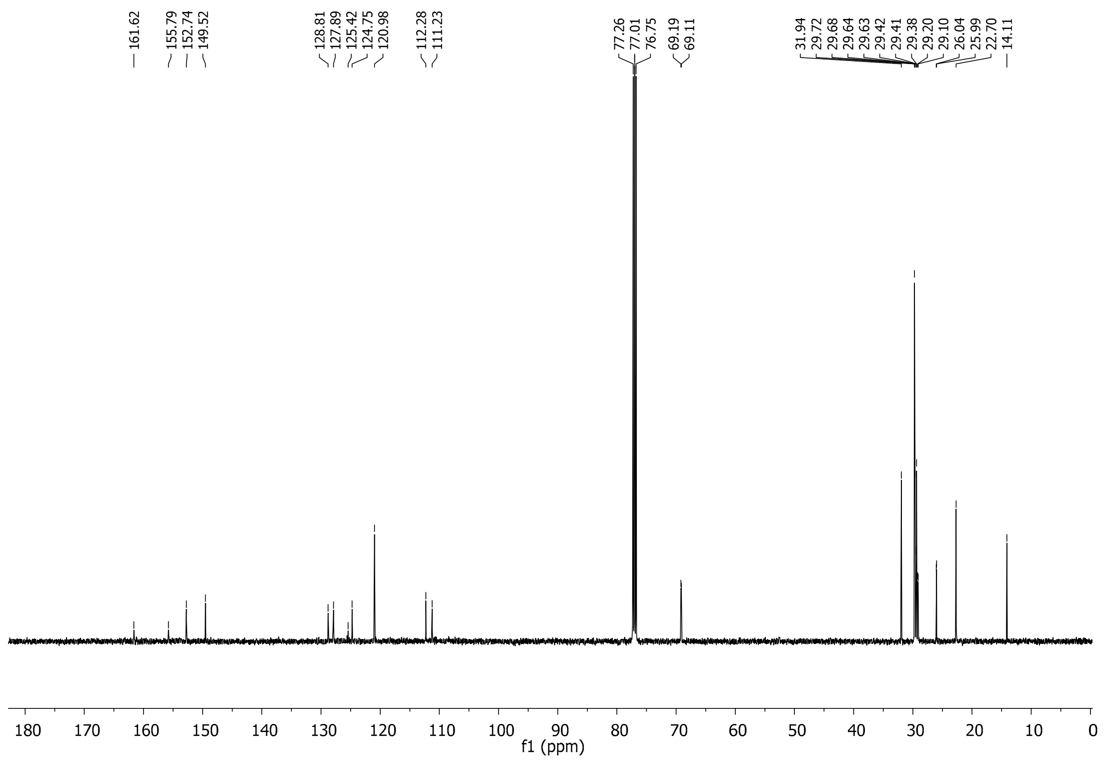

Figure S2. ^{13}C-NMR spectrum for compound **2**.

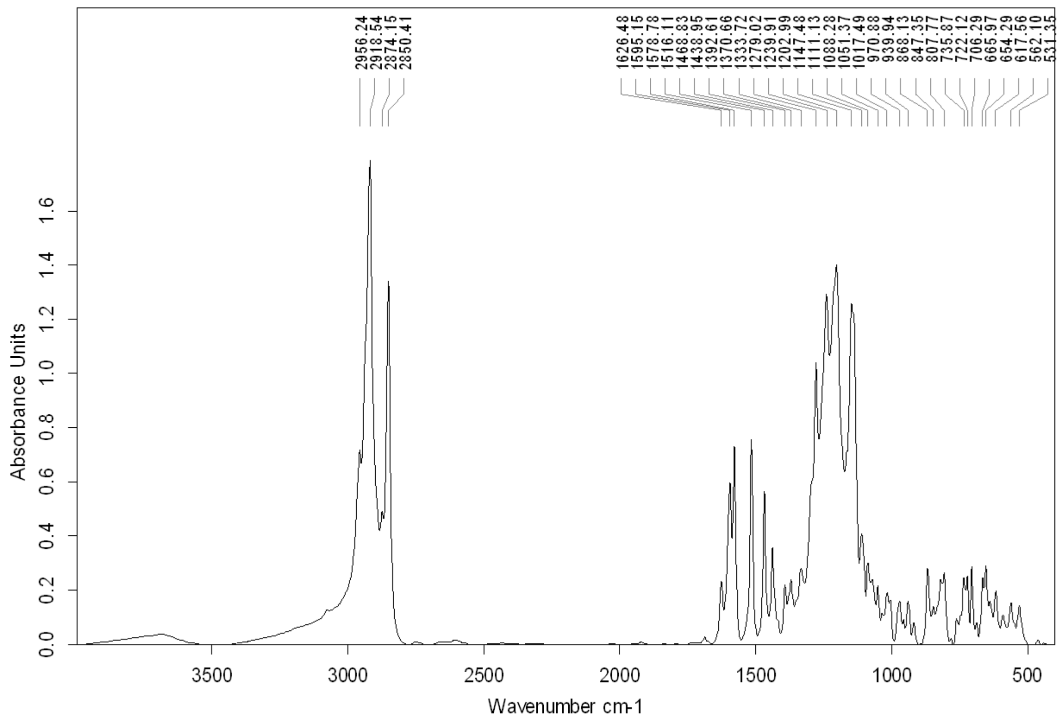

Figure S3. IR spectrum for compound **2**.

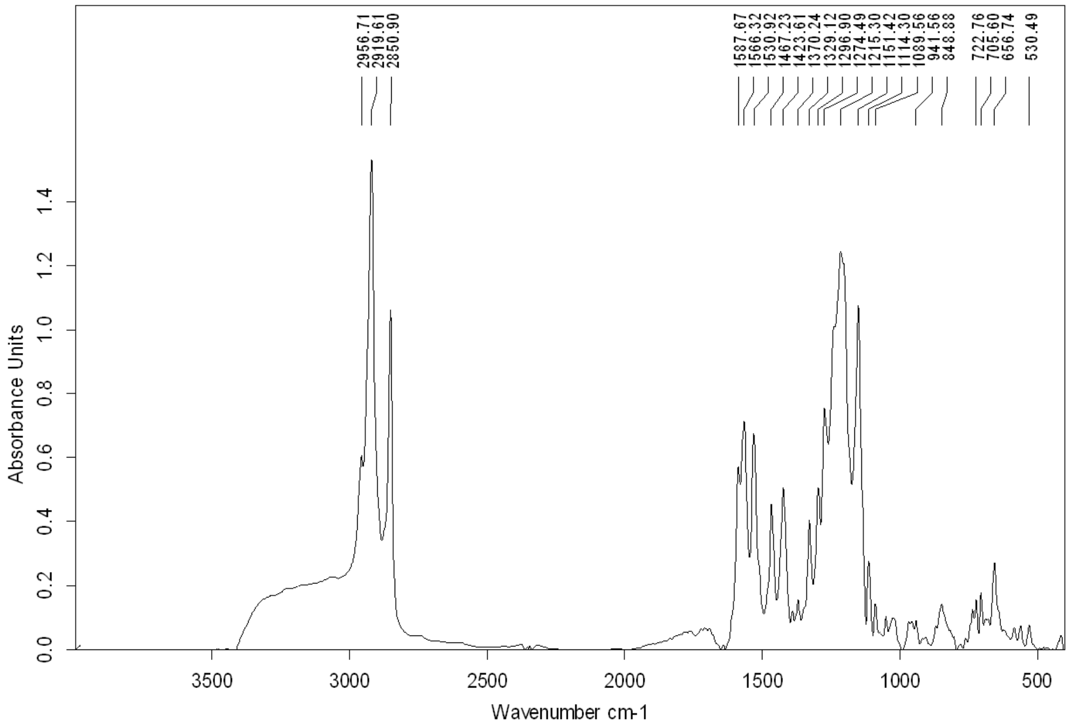

Figure S4. IR spectrum for compound **3**.

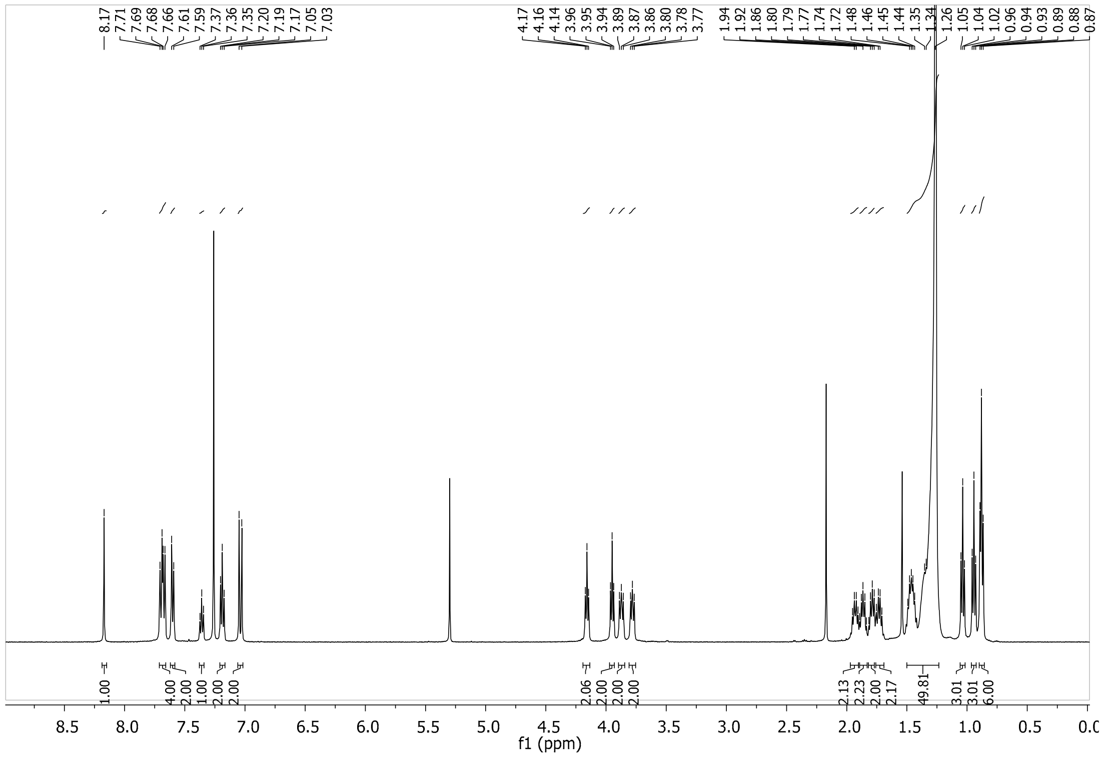

Figure S5. ^{1}H-NMR spectrum for compound **4-Pr**.

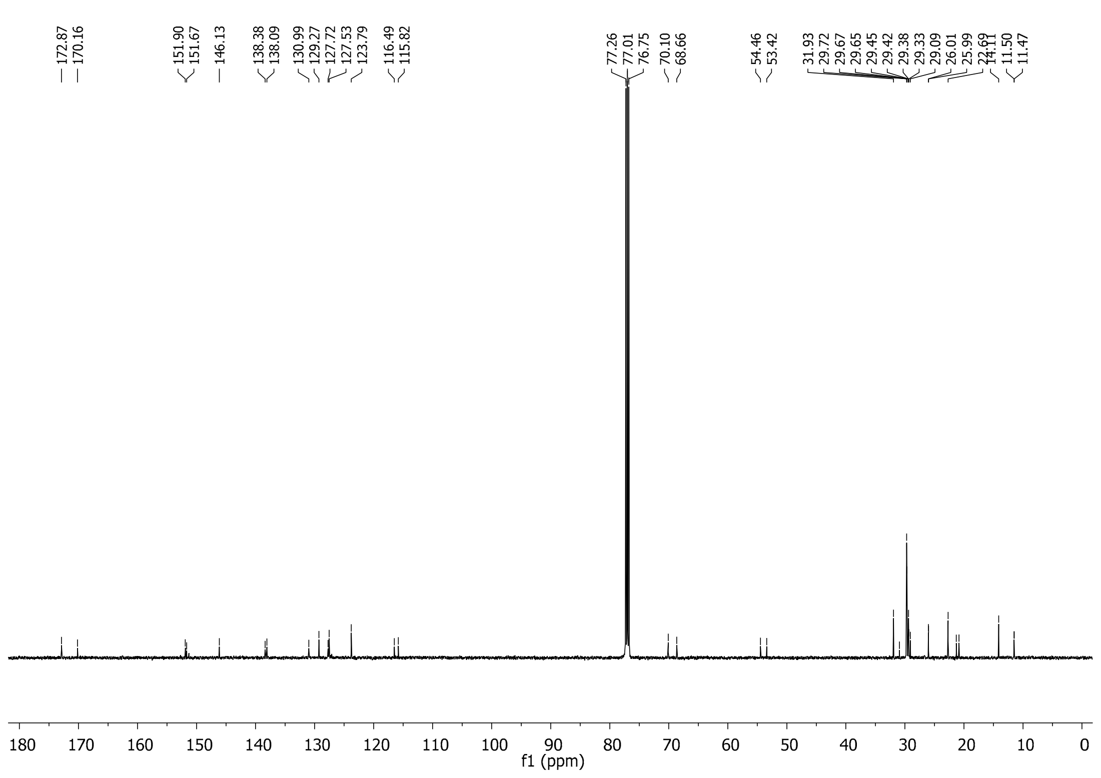

Figure S6. ^{13}C-NMR spectrum for compound **4-Pr**.

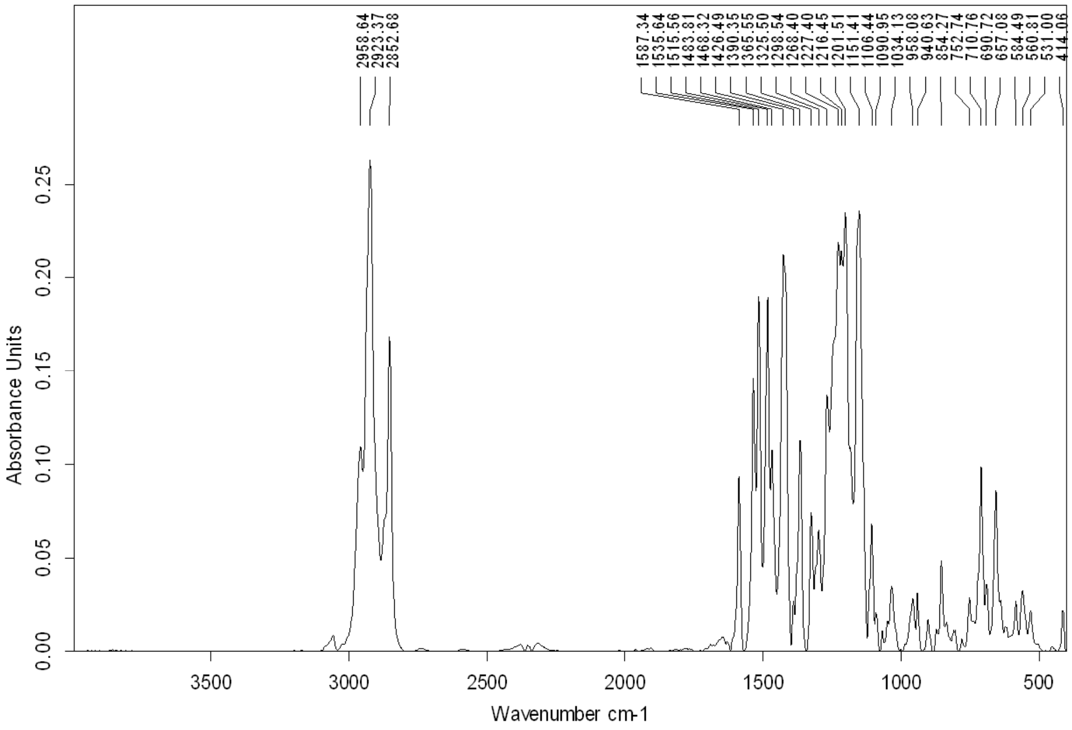

Figure S7. IR spectrum for compound **4-Pr**.

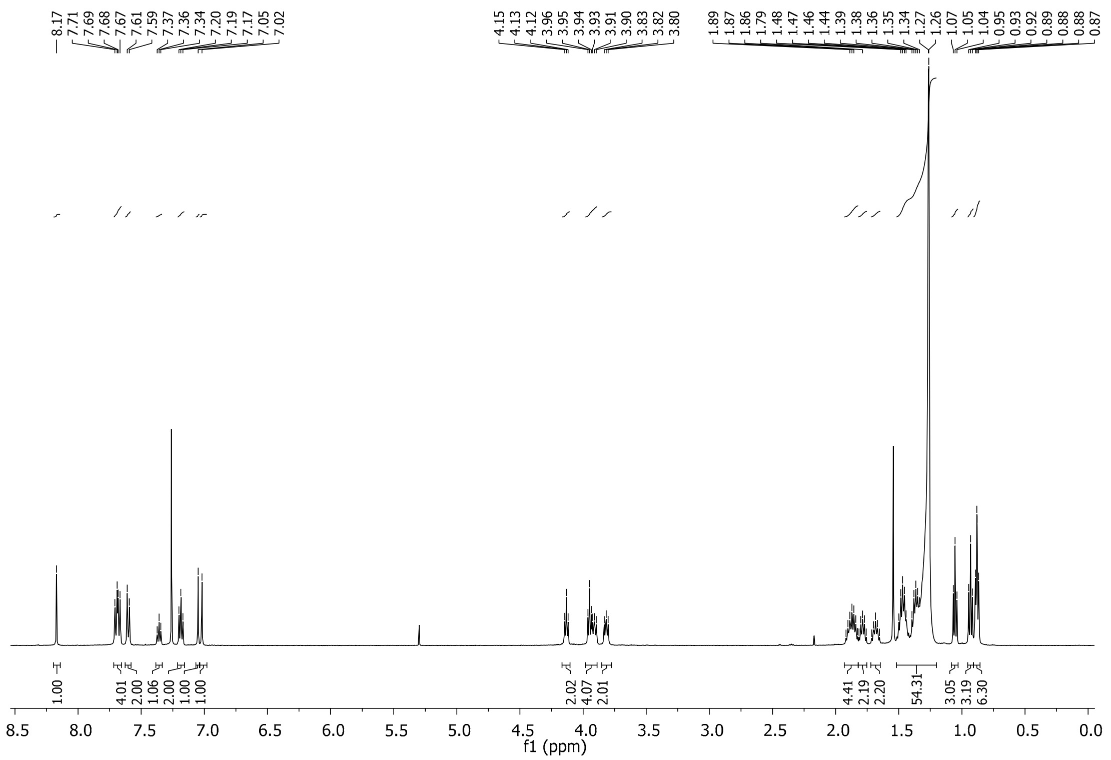

Figure S8. ^{1}H-NMR spectrum for compound **4-Bu**.

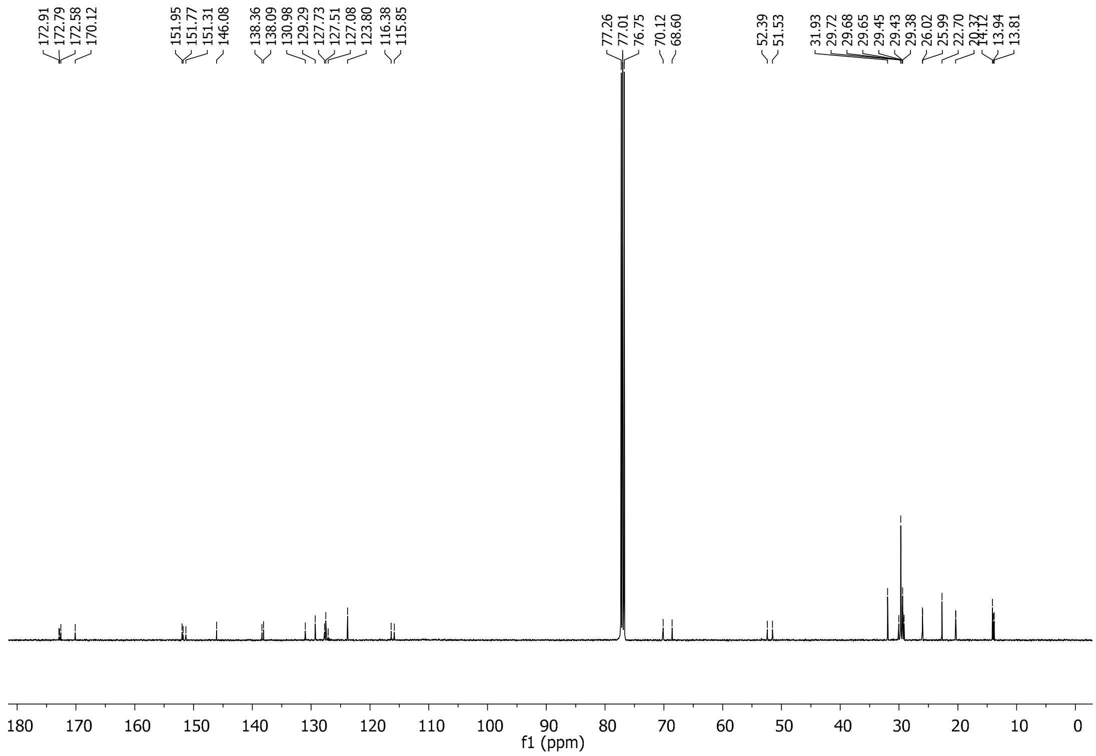

Figure S9. ^{13}C-NMR spectrum for compound **4-Bu**.

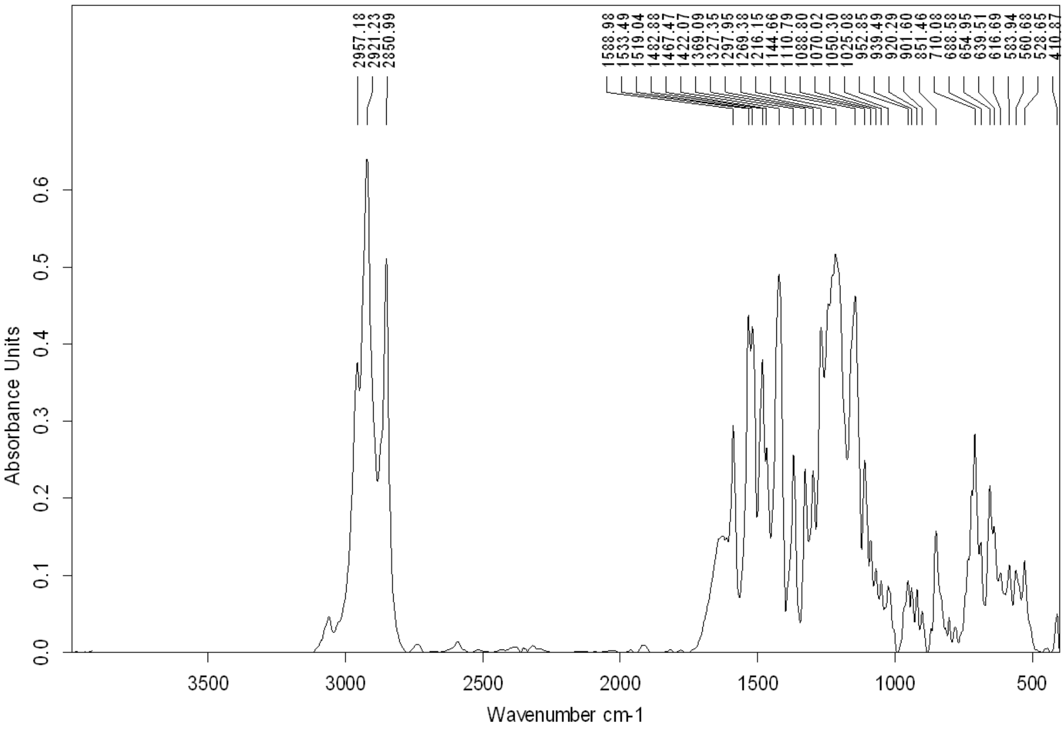

Figure S10. IR spectrum for compound **4-Bu**.

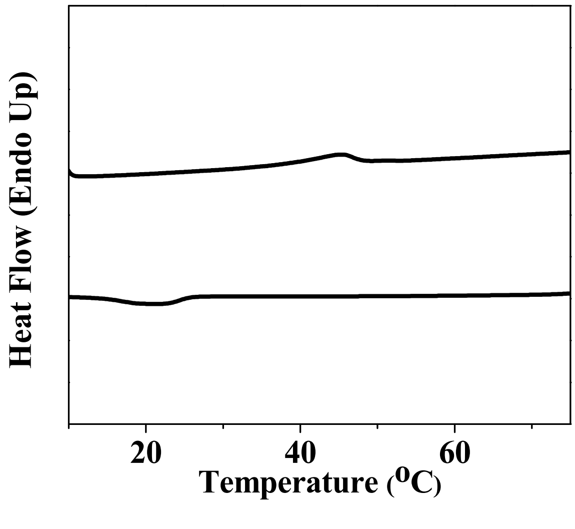

Figure S11. DSC trace for **4-Bu** 5% in 1-decanol.

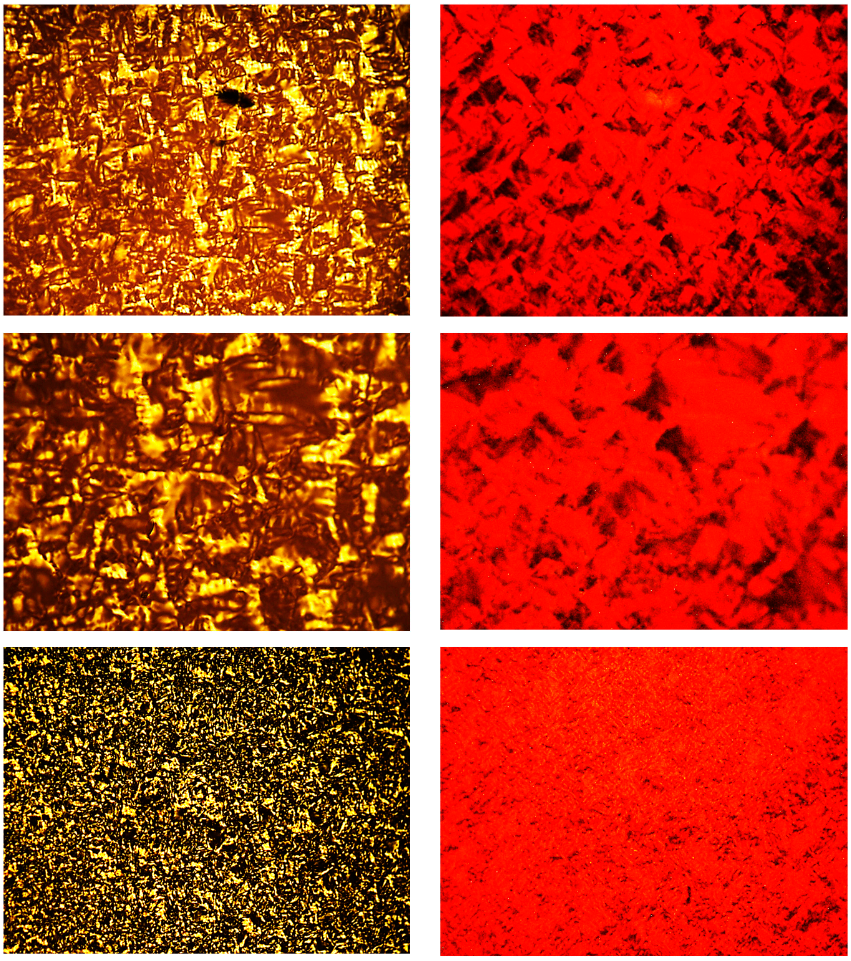

30 °C

35 °C

45 °C

(a) (b)

Figure S12. Lyotropic liquid crystal phase of compound **4-Pr** (15% gel) (a) under normal light and (b) under UV light at different temperatures.

Table S1. Crossover points, relationships between the viscoelastic moduli and dynamic viscosities (at 10 Hz) for the compounds **4-Pr** and **4-Bu** (dissolved in 1-decanol).

**t, ^{0}C**	**Sample**							
	**4-Pr in 1-decanol (50 mg/mL)**				**4-Bu in 1-decanol (50 mg/mL)**			
	**Heating**		**Cooling**		**Heating**		**Cooling**	
	**COP, Hz**	**Obs.**	**COP, Hz**	**Obs.**	**COP, Hz**	**Obs.**	**COP, Hz**	**Obs.**
**5**	-	G’≈G” (η_{dyn}=1.08 Pa·s at 10 Hz)	-	G’>G” (η_{dyn}=2.69 Pa·s at 10 Hz)	~2	G’>G” at f>COP (η_{dyn}=5.27 Pa·s at 10 Hz)	-	G’>G” (η_{dyn}=1.97 Pa·s at 10 Hz)
**10**	-	G’≈G” (η_{dyn}=1.19 Pa·s	~1.5	G’>G” at f>COP	~1.5	G’>G” at f>COP	-	G’>G” (η_{dyn}=1.99 Pa·s

		at 10 Hz)		(η_{dyn}=3.36 Pa·s at 10 Hz)		(η_{dyn}=5.94 Pa·s at 10 Hz)		at 10 Hz)
**15**	-	G’>G” (η_{dyn}=1.20 Pa·s at 10 Hz)	-	G’>G” (η_{dyn}=3.59 Pa·s at 10 Hz)	~2	G’>G” at f>COP (η_{dyn}=5.47 Pa·s at 10 Hz)	-	G’>G” (η_{dyn}=1.99 Pa·s at 10 Hz)
**20**	-	G’>G” (η_{dyn}=1.64 Pa·s at 10 Hz)	~0.5	G’>G” at f>COP (η_{dyn}=3.55 Pa·s at 10 Hz)	~2.5	G’>G” at f>COP (η_{dyn}=5.65 Pa·s at 10 Hz)	-	G’>G” (η_{dyn}=1.95 Pa·s at 10 Hz)
**25**	~2	G’>G” at f>COP (η_{dyn}=1.85 Pa·s at 10 Hz)	~0.5	G’>G” at f>COP (η_{dyn}=3.49 Pa·s at 10 Hz)	~3	G’>G” at f>COP (η_{dyn}=5.66 Pa·s at 10 Hz)	-	G’>G” (η_{dyn}=1.99 Pa·s at 10 Hz)
**30**	~1	G’>G” at f>COP (η_{dyn}=2.16 Pa·s at 10 Hz)	~0.5	G’>G” at f>COP (η_{dyn}=3.37 Pa·s at 10 Hz)	~2	G’>G” at f>COP (η_{dyn}=5.49 Pa·s at 10 Hz)	-	G’>G” (η_{dyn}=1.94 Pa·s at 10 Hz)
**35**	~1.5	G’>G” at f>COP (η_{dyn}=2.69 Pa·s at 10 Hz)	-	G’>G” (η_{dyn}=3.36 Pa·s at 10 Hz)	~1	G’>G” at f>COP (η_{dyn}=4.81 Pa·s at 10 Hz)	~0.5	G’>G” at f>COP (η_{dyn}=1.85 Pa·s at 10 Hz)
**40**	~2	G’>G” at f>COP (η_{dyn}=2.84 Pa·s at 10 Hz)	-	G’>G” (η_{dyn}=3.41 Pa·s at 10 Hz)	~1	G’>G” at f>COP (η_{dyn}=4.28 Pa·s at 10 Hz)	~0.5	G’>G” at f>COP (η_{dyn}=2.01 Pa·s at 10 Hz)
**45**	~0.5	G’>G” at f>COP (η_{dyn}=2.91 Pa·s at 10 Hz)	~0.5	G’>G” at f>COP (η_{dyn}=3.43 Pa·s at 10 Hz)	-	G’>G” (η_{dyn}=4.34 Pa·s at 10 Hz)	~0.5	G’>G” at f>COP (η_{dyn}=2.26 Pa·s at 10 Hz)
**50**	~1	G’>G” at f>COP (η_{dyn}=2.77 Pa·s at 10 Hz)	~0.5	G’>G” at f>COP (η_{dyn}=3.46 Pa·s at 10 Hz)	-	G’>G” (η_{dyn}=2.89 Pa·s at 10 Hz)	~0.8	G’>G” at f>COP (η_{dyn}=2.91 Pa·s at 10 Hz)
**55**	~0.5	G’>G” at f>COP (η_{dyn}=2.70 Pa·s at 10 Hz)	~0.5	G’>G” at f>COP (η_{dyn}=3.51 Pa·s at 10 Hz)	-	G’>G” (η_{dyn}=1.65 Pa·s at 10 Hz)	~1	G’>G” at f>COP (η_{dyn}=3.03 Pa·s at 10 Hz)
**60**	-	G’>G” (η_{dyn}=2.55 Pa·s at 10 Hz)	~0.8	G’>G” at f>COP (η_{dyn}=3.58 Pa·s at 10 Hz)	-	G’>G” (η_{dyn}=2.03 Pa·s at 10 Hz)	~2	G’>G” at f>COP (η_{dyn}=1.85 Pa·s at 10 Hz)
**65**	~1	G’>G” at f>COP (η_{dyn}=2.49 Pa·s at 10 Hz)	~0.8	G’>G” at f>COP (η_{dyn}=3.59 Pa·s at 10 Hz)	~0.5	G’>G” at f>COP (η_{dyn}=1.27 Pa·s at 10 Hz)	-	G”>G’ (η_{dyn}=0.71 Pa·s at 10 Hz)
**70**	~1	G’>G” at f>COP (η_{dyn}=2.50 Pa·s	~0.8	G’>G” at f>COP (η_{dyn}=3.59 Pa·s	-	G”>G’ (η_{dyn}=0.55 Pa·s at 10 Hz)	~1.5	G”>G at f>COP (η_{dyn}=0.13 Pa·s

		at 10 Hz)		at 10 Hz)				at 10 Hz)
**75**	~5	G'>G" at f>COP (η_{dyn}=2.23 Pa·s at 10 Hz)	~1	G'>G" at f>COP (η_{dyn}=3.66 Pa·s at 10 Hz)	~3.5	G">G' at f>COP (η_{dyn}=0.09 Pa·s at 10 Hz)	-	G">G' (η_{dyn}=0.03 Pa·s at 10 Hz)
**80**	~15	G'>G" at f>COP (η_{dyn}=1.53 Pa·s at 10 Hz)	~6	G'>G" at f>COP (η_{dyn}=3.10 Pa·s at 10 Hz)	~5.5	G">G' at f>COP (η_{dyn}=0.02 Pa·s at 10 Hz)	~5.5	G">G at f>COP (η_{dyn}=0.02 Pa·s at 10 Hz)
**85**	-	G">G' (η_{dyn}=0.79 Pa·s at 10 Hz)	-	G">G' (η_{dyn}=0.87 Pa·s at 10 Hz)				
**90**	-	G">G' (η_{dyn}=0.12 Pa·s at 10 Hz)	-	G">G' (η_{dyn}=0.12 Pa·s at 10 Hz)				

COP – crossover point; f – frequency; η_{dyn}=dynamic viscosity; G'>G" – system exhibiting preponderantly gel-like behavior (PGLB); G">G' – system exhibiting preponderantly liquid-like behavior (PLLB)

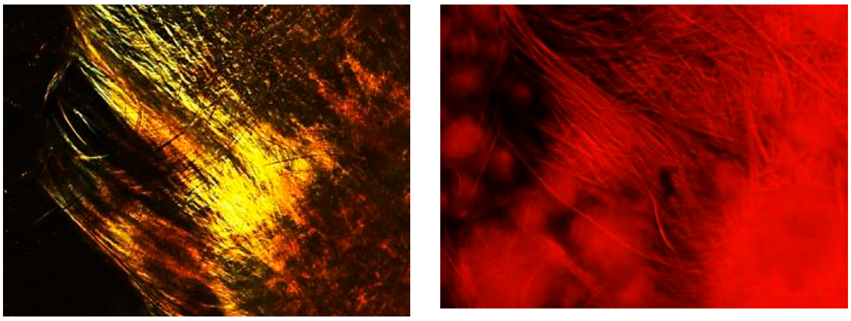

Figure S13. Gel morphology revealed by POM for compound **4-Pr** in (a) natural light and (b) under UV light (images taken during the gelation process at 25°C for DeOH-based gels containing 50 mg/mL).
